# Combination p53 activation and BCL-x_L_/BCL-2 inhibition as a therapeutic strategy in high-risk and relapsed acute lymphoblastic leukemia

**DOI:** 10.1038/s41375-024-02241-7

**Published:** 2024-04-10

**Authors:** Hayden L. Bell, Helen J. Blair, Samantha J. Jepson Gosling, Martin Galler, Daniel Astley, Anthony V. Moorman, Olaf Heidenreich, Gareth J. Veal, Frederik W. van Delft, John Lunec, Julie A. E. Irving

**Affiliations:** 1 Wolfson Childhood Cancer Research Centre, Newcastle University Centre for Cancer, Translational and Clinical Research Institute, Newcastle upon Tyne, UK; 2 Translational and Clinical Research Institute, Newcastle University Centre for Cancer, Newcastle upon Tyne, UK; 3 Princess Máxima Center for Pediatric Oncology, Utrecht, Netherlands

**Keywords:** Translational research, Preclinical research, Targeted therapies, Acute lymphocytic leukaemia

## Abstract

Due to the rarity of *TP53* mutations in acute lymphoblastic leukemia (ALL), p53 re-activation by antagonism of the p53-MDM2 interaction represents a potential therapeutic strategy for the majority of ALL. Here, we demonstrate the potent antileukemic activity of the MDM2 antagonist idasanutlin in high-risk and relapsed ex vivo coculture models of *TP53* wildtype ALL (*n* = 40). Insufficient clinical responses to monotherapy MDM2 inhibitors in other cancers prompted us to explore optimal drugs for combination therapy. Utilizing high-throughput combination screening of 1971 FDA-approved and clinically advanced compounds, we identified BCL-x_L_/BCL-2 inhibitor navitoclax as the most promising idasanutlin combination partner. The idasanutlin-navitoclax combination was synergistically lethal to prognostically-poor, primary-derived and primary patient blasts in ex vivo coculture, and reduced leukemia burden in two very high-risk ALL xenograft models at drug concentrations safely attained in patients; in fact, the navitoclax plasma concentrations were equivalent to those attained in contemporary “low-dose” navitoclax clinical trials. We demonstrate a preferential engagement of cell death over G_1_ cell cycle arrest, mechanistically implicating MCL-1-binding pro-apoptotic sensitizer NOXA. The proposed combination of two clinical-stage compounds independently under clinical evaluation for ALL is of high clinical relevance and warrants consideration for the treatment of patients with high-risk and relapsed ALL.

## Introduction

Despite the use of both intensive multi-modal cytotoxic therapeutic regimens and novel immune-based therapies, the prognosis for patients with relapsed/refractory acute lymphoblastic leukemia (ALL) remains poor. Less than ~50% of children and less than ~10% of adults with relapsed or refractory disease will experience long-term survival [1–5]. As such, the identification of novel therapeutic strategies for patients with relapsed/refractory ALL, irrespective of patient age, is clinically imperative.

The tumor suppressor protein p53, encoded by the *TP53* gene, plays a pivotal role in many fundamental processes and is critical to the maintenance of genomic integrity. Activated p53 bears a master regulatory function in the coordinated regulation of a plethora of transcriptional programs via interaction with key transcriptional coactivators and repressors; including genes involved in DNA repair, cell cycle progression, metabolic remodeling, and apoptosis [6]. Inactivation of p53 function is a frequent strategy employed by cancer cells to evade apoptosis [7]. Accordingly, *TP53* mutations are present in ~50% of solid tumors which drive oncogenesis by increasing cellular proliferation and survival [8]. Conversely, with an overall frequency of <10% at diagnosis and up to 30% at relapse, *TP53* mutations are relatively rare in ALL and particularly scarce in pediatric ALL [9–12]. Rather, alternative mechanisms often phenotypically compromise wild type p53 functions in the absence of loss-of-function *TP53* mutations such as alterations in MDM2, MDMX, and ARF — different regulators of p53.

As a transcriptional target of p53, the E3 ligase MDM2 tightly regulates the stability and localization of p53 in an autoregulatory feedback loop to prevent errant p53 activation under normal physiological circumstances. Consequently, oncogenic exploitation of the antagonistic properties of MDM2 is an appealing strategy by malignant cells to effectively abolish p53 function independent of *TP53* mutations. Indeed, *MDM2* overexpression is frequent and associated with poor outcomes in ALL [13]. Additionally, *CDKN2A* deletion — which encodes ARF, a p53 stabilizer via MDM2 antagonism — occurs in 30–50% of ALL, providing another means by which p53 function can be abrogated [14, 15]. As such, several agents have been developed which disrupt the p53-MDM2 interaction through binding to the MDM2 p53-binding pocket, in an effort to re-engage the tumor suppressor functions of p53. These agents are currently at various stages of clinical development [16]. Monotherapy with such MDM2 inhibitors, however, has shown modest clinical activity and some on-target dose-limiting toxicities leading to gastrointestinal complications and myelosuppression [17]. Consequently, combination strategies are likely required to obtain meaningful therapeutic benefits.

By using a synergism-focused, comprehensive drug combination screening approach in co-cultured patient and patient-derived xenograft ALL, we provide evidence for the potential therapeutic benefit of the combination of the MDM2 inhibitor idasanutlin and the BCL-x_L_/BCL-2 inhibitor navitoclax across a range of ALL subtypes, including high-risk and relapsed disease. While both drugs were highly potent apoptosis inducers in *TP53* wild type cells, their combination exerted strong synergy via a mechanism involving enhanced expression of the MCL-1 binding apoptosis sensitizer NOXA. Finally, the dual combination demonstrated significant inhibition of in vivo leukemia growth using high risk ALL xenograft models when compared to monotherapy or vehicle groups.

## Methods

### Human samples

Primary human ALL cells were density gradient-isolated (Lymphoprep, Stemcell Technologies, Cambridge, UK) from fresh and cryopreserved bone marrow aspirates of pediatric and adult patients (median age = 7.0 years) presenting or relapsing with ALL. Samples were accessed through the Newcastle Haematology Biobank, after appropriate informed consent in accordance with the Declaration of Helsinki (references 2002/111 and 07/H0906). PDX#11-r was kindly provided by O. Williams (UCL Great Ormond Street Hospital, London, UK). PDX#9-r was obtained from the Center for Patient Derived Models at Dana-Farber Cancer Institute (Boston, MA; sample DFAB-82241). Patient clinical details are given in Supplementary Table S1.

### Mouse experiments

NOD.Cg-Prkdc^scid^Il2rg^tm1Wjl^/SzJ (NSG) male and female mice were bred and housed under specific pathogen-free conditions at Newcastle University (Newcastle upon Tyne, UK) in individually ventilated cages with sterile bedding, water and diet (irradiated Teklad Global 19% protein extruded rodent diet 2919, Inotiv), with a 12 h light/dark cycle. Animal studies were approved by the Newcastle University Animal Welfare and Ethical Review Body and conducted in accordance with the Animals (Scientific Procedures) Act 1986 under the UK Home Office license *P74687DB5*.

### Patient-derived xenograft generation

Patient-derived xenografts (PDX) were generated as previously described [18] by intrafemoral injection of 0.5–1 × 10^6^ viable ALL cells in 8–12 weeks old NSG male and female mice under isoflurane anesthesia with provision of analgesia. Leukemia progression was monitored in the peripheral blood obtained from the tail vein by flow cytometry using red cell lysis and antibodies detailed in Supplementary Table S2. PDX cells were isolated from bone marrow, spleen, or liver (PDX#11-r only) of xenografted mice.

### In vitro drug treatment and assessment of cytotoxicity in a hTERT-immortalized MSC co-culture model

Primary and PDX ALL blasts drug sensitivity was determined in a coculture model using hTERT-immortalized bone marrow mesenchymal stem cells (MSCs) and live cell numbers were enumerated by fluorescence image analysis with machine learning as previously described [19]. For drug combinations, cells were treated with eight two-fold serial dilutions of each compound either individually or in all possible permutations in a checkerboard fashion. Drug effects were determined after four days of incubation at 37 °C/5% CO_2_.

### Flow cytometric analysis

Apoptosis was analyzed using an Annexin V Apoptosis Kit (556547, BD Biosciences). Cell cycle analysis by propidium iodide (50 μg/mL) staining with RNase A (20 μg/mL) was performed on cells fixed with 70% (v/v) ice-cold ethanol by flow cytometry. Data were analyzed using FlowJo software.

### Statistical analyses

Statistical analyses were performed with GraphPad Prism 9 (San Diego, CA, USA) software and Python programming language. Two-tailed *T*-tests assessed differences between two groups. One-way or two-way ANOVA with Tukey’s correction for multiple comparisons was used for analyses involving three or more groups. Data normality was assessed by Shapiro-Wilks test. *P*-values < 0.05 were considered statistically significant. Drug combination effects and synergy scores were analyzed using the Bliss independence model [20, 21]. No tests were used for in vitro sample size estimation.

Further methods can be found in Supplementary information.

## Results

### p53 activation by idasanutlin causes potent, on-target cytotoxic activity in *TP53*-wildtype ALL samples

Firstly, we determined whether the MDM2 inhibitor idasanutlin depends on wildtype p53 for its activity in ALL using an isogenic relapsed NALM6 cell line model with wildtype and monoallelic/biallelic *TP53* deletion (Supplementary Fig. S1A). In both p53-competent cell lines, idasanutlin exposure potently decreased cell viability (Supplementary Fig. S1B). Supporting on-target idasanutlin activity, we observed robust p53 accumulation and upregulation of the p53 transcriptionally-regulated target gene products MDM2 and p21 (Supplementary Fig. S1C), marked dose-dependent G_1_-phase cell cycle arrest (Supplementary Fig. S1D), and increased apoptosis induction (Supplementary Fig. S1E). In contrast, idasanutlin did not trigger such changes in p53^null^ cells. These results demonstrate that idasanutlin can effectively activate p53 and induce apoptosis in a p53-dependent manner.

**Fig. 1:**
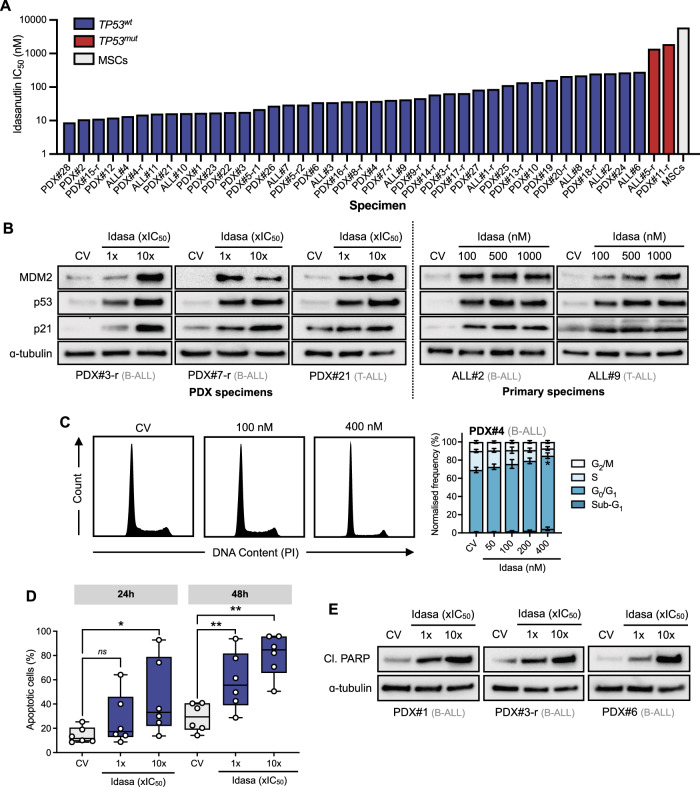
p53 activation by idasanutlin exhibits potent, on-target cytotoxic activity in *TP53*-wildtype high-risk and relapsed ALL samples. **A** Sensitivity of primary (*n* = 11) and patient-derived xenograft (*n* = 31) ALL samples to idasanutlin exposure for 96 h in ex vivo co-culture with hTERT-immortalized MSCs. Drug responses were determined by fluorescence image-based microscopy in at least technical duplicate, relative to respective vehicle (DMSO) control. IC_50_ values are based on live cell enumeration. (*N* = 1) **B** Immunoblots showing MDM2, p53, and p21 expression following exposure to respective idasanutlin IC_50_ fractions for 6 h (*N* = 1). **C** Cell cycle analysis of B-ALL PDX#4 exposed to increasing concentrations of idasanutlin for 24 h. Error bars show mean ± s.d. of *N* = 3 independent experiments. **D** Apoptotic cells were quantified by annexin-V flow cytometry in PDX cells following exposure to respective idasanutlin IC_50_ fractions for 24 and 48 h (*N* = 1 for each respective sample). Error bars show mean±s.d. **E** Immunoblots showing induction of cleaved PARP (Asp214) following 24 h exposure to respective idasanutlin IC_50_ fractions (*N* = 1). In (**B**, **E**) ɑ-tubulin served as the loading control.

Next, we investigated the antileukemic effects of idasanutlin in a cohort of primary (*n* = 11) and primary-derived (PDX, *n* = 31) B- (*n* = 30) and T-ALL (*n* = 12) samples derived from pediatric and adult ALL patients representing various subtypes including *KMT2A*-rearranged, Ph^+^, *TCF3::HLF*, and low hypodiploidy ALL (Supplementary Table S1). We utilized an ex vivo coculture model of ALL blasts on hTERT-immortalized mesenchymal stem cells (MSCs) to support short-term leukemic blast survival and growth, complemented by a fluorescence image-based microscopy platform. Idasanutlin exerted potent, dose-dependent antileukemic activity in the large majority of cases (*n* = 40/42) with a mean±s.d. IC_50_ of 76 ± 84 nM (Fig. 1A) which is well below clinically achievable concentrations after oral administration of idasanutlin (C_max_ = 3–9 μM) (refs. [17, 22, 23]). The two exceptions harbored homozygous inactivating *TP53* mutations (Supplementary Table S3). There was no differential idasanutlin sensitivity between presentation and relapse samples (*p* = 0.956) or between B- and T-ALL lineage (*p* = 0.832)(Supplementary Fig. S2A). In contrast, non-leukemic bone marrow MSCs were unaffected by idasanutlin activity at concentrations effective in leukemic cells; with effects only observed at approximately 80-fold higher concentrations (Fig. 1A). Consistent with cell line data, idasanutlin exposure robustly enhanced protein expression of p53, MDM2, and p21 in *TP53* wild type samples, validating effective engagement of the p53 pathway (Fig. 1B). Additionally, basal MDM2 protein expression did not correlate with idasanutlin sensitivity (Supplementary Fig. S3). Using high-risk *TCF3::HLF*-rearranged sample PDX#4, idasanutlin exposure induced dose-dependent G_0_/G_1_ phase cell cycle arrest by 24 h (Fig. 1C). To confirm engagement of cell death in response to idasanutlin, Annexin V staining demonstrated a time-dependent 30 ± 15% increase in apoptosis amongst PDX samples (*n* = 6) exposed to their respective IC_50_ concentrations of idasanutlin for 48 h (*p* = 0.004; Fig. 1D). Apoptotic cleaved PARP expression levels were markedly enhanced following 24 h exposure to idasanutlin, corroborating the induction of apoptotic cell death (Fig. 1E).

Together, these results emphasize the antileukemic potential of p53 re-engagement by idasanutlin as a therapeutic strategy against a broad range of ALL subtypes harboring wildtype *TP53* at concentrations well-below clinically attainable levels.

### Combination drug screening of 1971 FDA-approved and/or clinically advanced compounds identifies synergistic candidate idasanutlin combinations against high-risk ALL

**Fig. 2:**
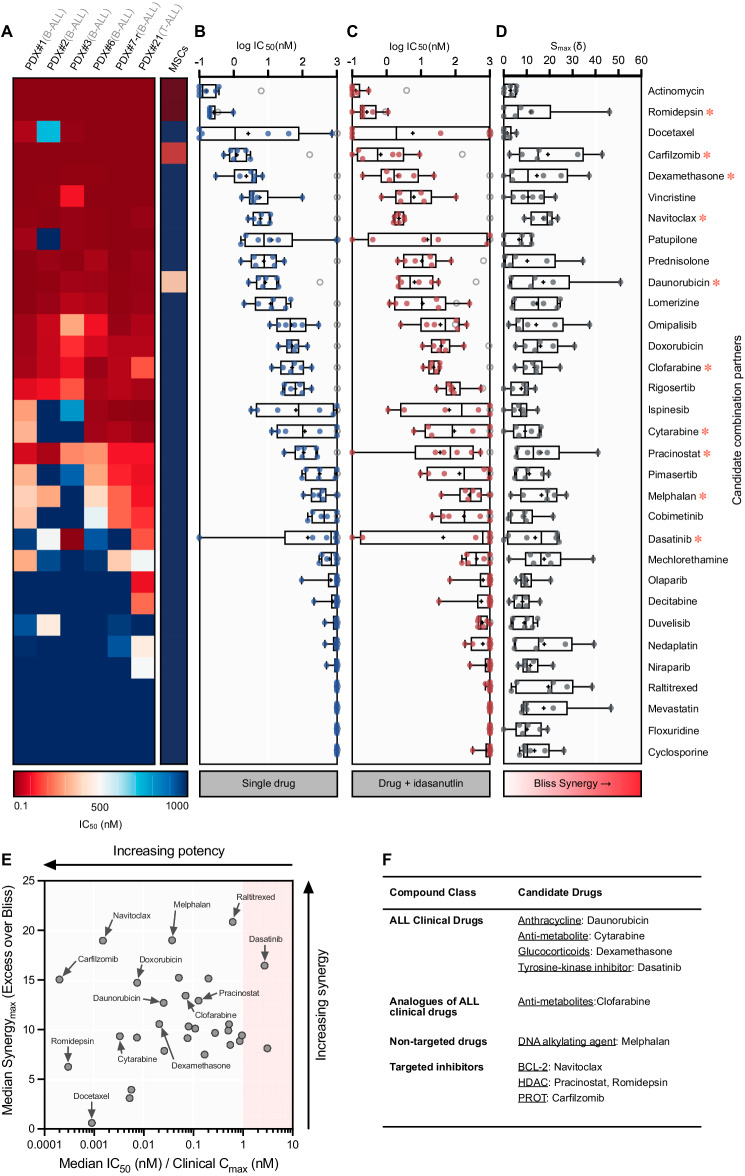
Combinatory high-throughput drug repurposing library screening with 1971 FDA-approved drug compounds identifies potential synergistic idasanutlin combination partners. **A** Heat map depicting drug concentrations resulting in 50% cell death (IC_50_) of 32 combination candidates determined using a 5-point log_10_-fold dilution range in a panel of 6 PDX leukemic samples. Each column represents an individual leukemia PDX sample, and each row represents a unique drug. Mean cell viability was determined in technical triplicate after 96 h drug exposure using fluorescence image-based microscopy (*N* = 1). Absolute live cell counts were normalized to respective vehicle (DMSO) controls. Drugs chosen for further investigation are indicated by a red asterisk (). **B** Box plot showing the distribution of mean IC_50_ values across each sample (*N* = 1). Mean MSC IC_50_ is indicated as a grey ring (*N* > 3) or bound to the highest concentration if > 1 μM. Boxes represent 25th to 75th percentiles, with median as a solid line, mean as a ‘+’, and whiskers extending to the range. **C** Box plot showing the mean IC_50_ for each PDX sample exposed to each library drug in combination with their respective idasanutlin doses resulting in approximately 40% cell death (IC_40_). Data were normalized to idasanutlin-treated cells to specifically examine the additive effects of the library drugs. **D** Distribution of maximum attained excess over Bliss synergy scores (S_max_) for each drug in combination with respective idasanutlin IC_40_ and IC_60_ concentrations. **E** Dot plot showing the median IC_50_ of all PDX as a fraction of the clinically-reported *C*_max_ for the respective drugs versus the median S_max_ score of all PDX samples. Clinically-reported pharmacokinetics and sources are provided in Supplementary Table S7. The red-shaded region indicates drugs with a median IC_50_ greater than clinically-reported limits. Results for mechlorethamine and mevastatin were omitted as clinical pharmacokinetic properties are unavailable. **F** Most promising drug combination candidates selected for further investigation.

To identify drug combinations which enhance idasanutlin activity in ALL, we first performed high-throughput screening against 1971 clinically advanced or FDA-approved compounds using the relapsed (*TP53*^wt^) NALM6 cell line (Supplementary Fig. S4, Supplementary Tables S4–6). The topmost potent and/or synergistic candidates were examined in a panel (*n* = 6) of PDX samples — including relapsed and poor outcome-associated subtypes — in coculture with MSCs. The majority of drugs (*n* = 23/32) demonstrated sub-micromolar potency across the PDX panel (> 50%), including each investigated conventional ALL drug (*n* = 6) (Fig. 2A, B). To characterize potential idasanutlin combination effects, candidates were combined with idasanutlin at two sub-lethal concentrations for each respective PDX specimen (Fig. 2C) and drug interactions were evaluated (Fig. 2D). Overall, significant synergistic activity (median synergy max (S_max_) > 10) was observed in 16/32 combination candidates.

To maximize clinical translation of identified idasanutlin combination candidates and further select for the most promising candidates, we considered drug potency in the context of the safely-achievable plasma *C*_max_ for each drug in human studies (Fig. 2E, Supplementary Table S7). Candidates (*n* = 10) were selected for further validation based upon highest ranking drug synergy and potency with respect to reported safely achievable clinical concentrations (Fig. 2F).

### Idasanutlin synergistically enhances the antileukemic effects of multiple FDA-approved and clinically advanced drug classes

**Fig. 3:**
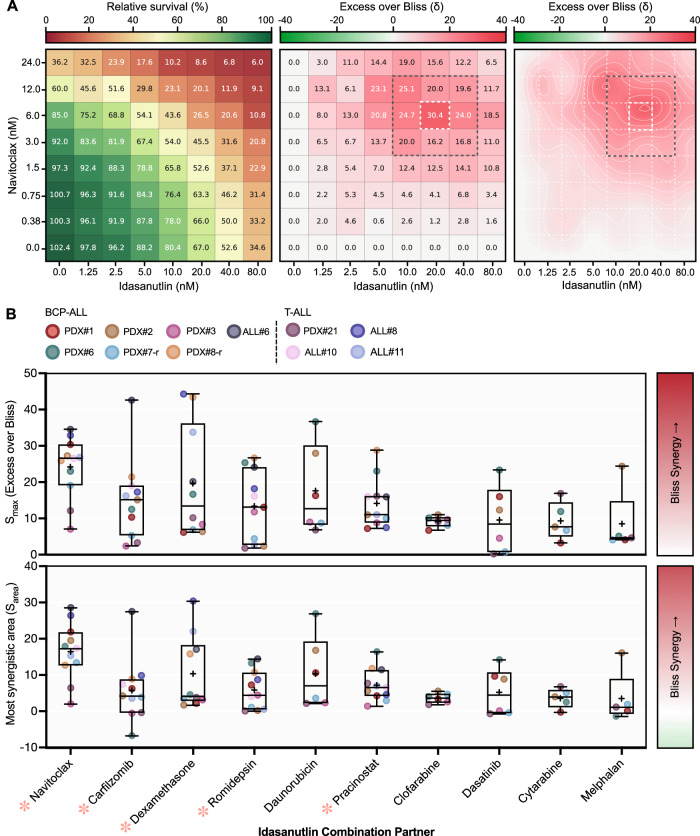
Idasanutlin enhances the antileukemic effects of multiple FDA-approved and clinically advanced drug classes. **A** Representative pairwise combination of idasanutlin with navitoclax in B-ALL PDX#1. Cells were subjected to increasing doses of idasanutlin or navitoclax alone, or a combination of the two simultaneously for 96 h, and viability was assessed using fluorescence image-based microscopy. *N* = 1 independent experiments in duplicate. Heat map (left) represents mean viability relative to vehicle (DMSO) control. Excess over Bliss synergy scores (center) were calculated at each dose combination (< 0, antagonism; 0, additive; > 0 synergy). Two-dimensional contour plots (right) represent the drug interaction landscape for the indicated combination concentrations derived from spline interpolation of the synergy scores. Dark gray dashed box indicates the most synergistic area with the S_area_ derived from the mean of these values. White dashed box indicates synergy max (S_max_). **B** Maximum Excess over Bliss synergy scores and most synergistic area Bliss synergy scores (S_area_) for each candidate idasanutlin drug combination. Data are ordered by median S_max_ score. *N* = 1 independent experiments in duplicate. Drug combination candidates selected for additional validation are indicated with a red asterisk (). Synergy scores for cytarabine and melphalan in PDX#3 were omitted due to single-agent resistance. Box plots: boxes, 25th to 75th percentile; midline, median; ‘+’ symbol, mean; whiskers, data range.

The highest ranking idasanutlin combination candidates were examined more extensively using a low-throughput methodology. First, we validated the synergistic combination interaction of the candidate drugs (*n* = 10) with idasanutlin in the same panel of ALL samples used for the screen. Individualized drug doses were utilized in an 8-by-8 combination matrix to incorporate a broad range of intermediate effects for each respective drug and PDX specimen (Fig. 3A). Drug combination activity was quantified using two metrics of Bliss synergy — S_max_ and most synergistic area score (S_area_) — with all candidates attaining at least additive activity with idasanutlin (Fig. 3B). Moreover, counter-screening of each candidate in isogenic p53^wt^ and p53^null^ NALM6 cells invariably confirmed on-target p53-dependent activity of each drug combination pair (*p* < *0.05*; Supplementary Fig. S5). We extended our analyses and evaluated combination activity of the top five synergy-ranked drugs in an extended panel of ALL samples (*n* = 5; Fig. 3B). The top ranked candidates in our screen were: navitoclax, carfilzomib, dexamethasone, romidepsin, and pracinostat. Exception was made to combination with daunorubicin due to concerns of potentially elevated cardiotoxicity in heavily pre-treated relapsed ALL patients [24, 25].

Overall, our comprehensive drug screening approach identified the combination of idasanutlin and navitoclax to provide both the greatest and most consistent synergistic interaction (S_max_ = 24.2 ± 8.4, *n* = 11) of the candidate combinations across a broad landscape of dose combinations.

### Combined p53 activation and targeting of BCL-x_L_/BCL-2 induces potent and synergistic apoptosis in primary and primary-derived ALL samples

**Fig. 4:**
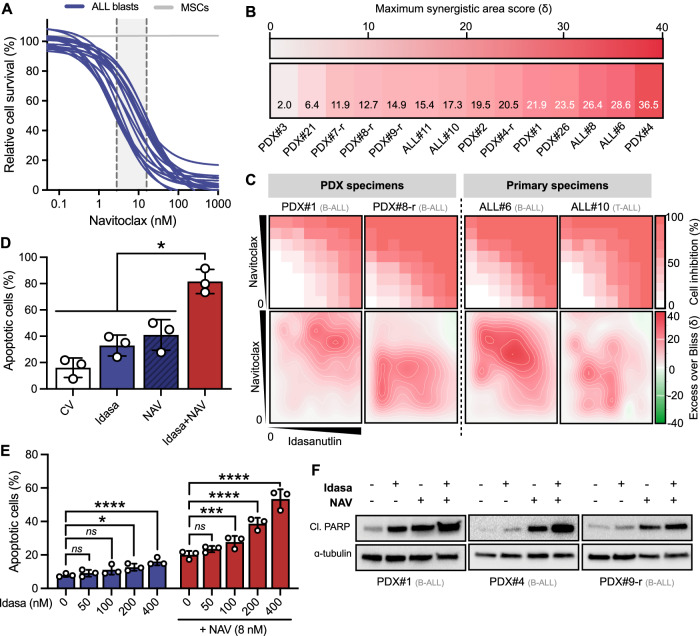
Idasanutlin in combination with BCL-x_L_/BCL-2 inhibitor navitoclax exhibits significant ex vivo efficacy against high-risk ALL xenograft and patient samples. **A** Sensitivity of primary (*n* = 4) and PDX (*n* = 13) leukemic cells exposed to increasing concentrations of NAV (0.1–1000 nM) for 96 h in ex vivo co-culture with hTERT-immortalized MSCs. Drug responses were determined by fluorescence image-based microscopy in at least technical duplicate and normalized to respective vehicle (DMSO) controls. IC_50_ values are based on live cell enumeration. *N* = 1 independent experiment in at least duplicate. The gray boundary indicates the IC_50_ range. **B** Heat map of most synergistic area Bliss synergy scores (S_area_) for idasanutlin with navitoclax combination activity in primary (*n* = 4) and PDX (*n* = 10) ALL samples ex vivo. Leukemic samples included relapse patients and patients with subtypes linked to unfavorable survival. *N* = 1 independent experiments in at least duplicate. **C** Representative dose-response matrix analyses showing cell inhibition (top) and synergistic landscape (bottom) across diverse idasanutlin-navitoclax dose combinations. **D** Apoptotic cells were quantified by annexin-V flow cytometry in PDX samples (*n* = 3) following exposure to respective IC_50_ concentrations of idasanutlin, navitoclax, or their combination for 48 h. Error bars show mean ± s.d. Combination treated cells were compared to respective single drugs by paired *t-*tests. **E** Apoptotic cells were quantified by annexin-V flow cytometry in PDX#4 cells following exposure to indicated concentrations of idasanutlin ± navitoclax (8 nM) for 24 h (*n* = 3). Error bars show mean ± s.d. Data were analyzed by two-way ANOVA with Tukey posttest for multiple comparisons. **F** Immunoblots showing induction of cleaved PARP (Asp214) in PDX samples (*n* = 3) following exposure to respective IC_50_ concentrations of idasanutlin, navitoclax, or their combination for 24 h. ɑ-tubulin served as the loading control. ns=not significant, *p value < 0.05, ***p value < 0.001, ****p value < 0.0001.

BCL-x_L_/BCL-2 inhibitor navitoclax consistently showed potent in vitro activity against an extended panel of primary and PDX ALL samples (mean IC_50_ = 7.8 ± 4.6 nM, *n* = 17 Fig. 4A); concentrations well below clinically reported plasma levels following oral administration of navitoclax in humans (*C*_max_ = 4–6 μM) [26, 27]. We did not observe any differential navitoclax sensitivity between presentation and relapse (*p* = 0.198) or B- and T-lineage samples (*p* = 0.510) (Supplementary Fig. S2B). We further extended our sample panel to validate idasanutlin-navitoclax efficacy in additional ALL subtypes. Overall, co-treatment with idasanutlin-navitoclax was highly synergistic across 12/14 tested specimens including many high-risk and relapsed ALL subtypes (average Bliss S_area_ = 18.4 ± 8.7, *n* = 14), indicating that the synergistic effect is not restricted to a single ALL subtype or lineage (Fig. 4B). Furthermore, extensive regions of additive through to highly synergistic combination effects were evident in the drug combination dose-interaction landscapes, indicating synergy across a broad range of dose-combination ratios (Fig. 4C, Supplementary Fig. S6). Conversely, there was no evidence of any synergistic drug interaction in the bone marrow-derived MSCs at concentrations effective in leukemic cells (S_area_ = 3.6 ± 0.9; Supplementary Fig. S7). The synergistic effects of p53 activation with navitoclax were recapitulated with the clinically relevant alternative and structurally-unrelated MDM2 inhibitors siremadlin (HDM201) and navtemadlin (AMG-232) using three high-risk PDX samples (Supplementary Fig. S8).

Given that navitoclax triggers intrinsic mitochondrial apoptosis [28], we investigated the effects of idasanutlin-navitoclax co-exposure on apoptosis induction. While the proportion of apoptotic cells was efficiently enhanced in response to either drug alone in relapsed cell lines (Supplementary Fig. 9A) and PDX samples (Fig. 4D), this was significantly increased upon co-exposure to both drugs (*p* < 0.05). In very high-risk B-lineage PDX#4, combination death significantly increased in a dose-dependent manner as compared to monotherapies (Fig. 4E). These findings were further corroborated by enhanced levels of cleaved PARP in response to the drug combination as compared to monotherapies (Supplementary Fig. 9B; Fig. 4F).

### The idasanutlin-navitoclax combination preferentially engages cell death over cell cycle arrest and pro-apoptotic protein NOXA is mechanistically implicated

**Fig. 5:**
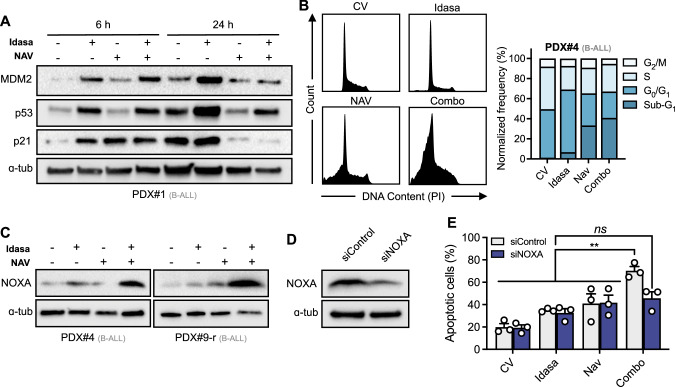
Combination of idasanutlin and navitoclax prioritizes cell death over cell cycle arrest via a mechanism involving pro-apoptotic protein NOXA. **A** Representative immunoblots showing MDM2, p53, and p21 expression in B-ALL PDX#1 following exposure to respective IC_50_ concentrations of idasanutlin, navitoclax, or their combination for 6 h or 24 h (*N* = 1). **B** Cell cycle analysis of B-ALL PDX#4 exposed to respective IC_50_ concentrations of idasanutlin, navitoclax, or their combination for 24 h. *N* = 1 independent experiment. **C** Immunoblots showing protein expression levels of NOXA in PDX samples following exposure to respective IC_50_ concentrations of idasanutlin, navitoclax, or their combination for 24 h. Data show representative data of *N* = *3* independent PDX samples. **D** Immunoblot showing representative protein expression of NOXA/PMAIP1 following siRNA-mediated (300 nM) knockdown of *NOXA/PMAIP1* or a non-targeted control in the NALM6 cell line. *N* = 3 independent experiments. **E** Apoptotic cells were quantified by annexin-V flow cytometry in NALM6 cells transfected with non-targeting control siRNA or *PMAIP1/NOXA* siRNA, following exposure to respective IC_50_ concentrations of idasanutlin, navitoclax, or their combination for 48 h. Error bars show mean ± s.d of three independent experiments. Data were analyzed by two-way ANOVA with Tukey posttest for multiple comparisons. ns=not significant, **p-value < 0.01. In (**A**, **C**, **D**), ɑ-tubulin served as the loading control.

To gain insight into the mechanism(s) responsible for the superior antileukemic activity of the idasanutlin-navitoclax combination, we first investigated p53 signaling pathway activation using three high-risk B-ALL PDX samples. As anticipated, exposure to idasanutlin resulted in robust p53 protein accumulation with corresponding increased levels of MDM2 and p21 at both 6- and 24-hour time points (Fig. 5A). Despite evidence of comparable p53 activation in combination-treated cells at 6 h, combination-treated cells demonstrated significantly lower p53 levels at 24 h, with corresponding decreases in MDM2 and p21 levels as compared to idasanutlin-only treated cells. Given this significant reduction of p21 and the clear enhancement of cell death in combination exposed cells (Fig. 4D–F), these data suggest p53 activation with concurrent BCL-x_L_/BCL-2 inhibition redirects the outcome of p53 activation from cell cycle arrest, conducive of cell survival, to apoptosis. In support, monotherapy idasanutlin exposure induced substantial G_1_-phase cell cycle arrest while co-treatment with navitoclax promoted a substantial proportion of cells with sub-G_1_ DNA content (Fig. 5B), consistent with a prioritization of apoptotic cell death over cell cycle arrest.

Next we investigated downstream effectors of p53-dependent apoptosis by evaluating pro- and anti-apoptotic BCL-2 family member protein levels using the relapsed cell lines NALM6 and RS4;11 as models (Supplementary Fig. S10A). Anti-apoptotic MCL-1 levels were increased in response to navitoclax and combination drug treatments, a well-documented resistance factor to BCL-2 family inhibitors [29, 30]. In contrast to studies in AML [31], we did not observe a decrease in MCL-1 levels in response to idasanutlin alone. Interestingly, we observed a strong up-regulation of the MCL-1 binding and p53 transcriptionally-regulated target protein NOXA, but not PUMA, in combination treated cells as compared to single drugs at these time points, seen most strongly at 24 h in both cell lines (*p* < 0.05) and corroborated by enhanced *PMAIP1* (encoding NOXA) mRNA expression (Supplementary Fig. S10B). In support, NOXA protein levels were substantially increased in combination-exposed PDX at 24 h (*n* = 3, Fig. 5C). Efficient siRNA-mediated knockdown of NOXA (Fig. 5D) severely compromised synergistic interaction of idasanutlin and navitoclax, significantly reducing apoptotic response to the combination but not single drug responses (Fig. 5E). These data suggest that NOXA is functionally implicated in the enhanced cell death mediated by idasanutlin-navitoclax combination treatment.

### Idasanutlin and navitoclax combination significantly decreases leukemia burden over monotherapy in high-risk in vivo xenograft ALL models

Next we assessed the therapeutic potential of combined p53 activation and BCL-x_L_/BCL-2 inhibition by evaluating the ability of idasanutlin-navitoclax to inhibit growth of patient leukemia cells in vivo. To ensure the use of oral doses of both drugs achieving lower than clinically-attainable plasma levels in human patients, we determined steady state (day 3) drug plasma concentrations in highly-immunodeficient NSG mice (Supplementary Table S8) at murine doses far within range of human equivalent doses [32]. The steady-state plasma concentration of idasanutlin (30 mg/kg QD) was 2.81 ± 1.39 μM (*n* = 3) and navitoclax (50 mg/kg QD) was 0.91 ± 0.66 μM (*n* = 3). Co-administration of both drugs did not significantly change their respective plasma concentrations, discrediting potential pharmacokinetic drug-drug interactions (*p* > 0.05). A preliminary toxicity study demonstrated the combination treatment to be well tolerated (*n* = 5) with no adverse effects on overall health, survival, or average body weight over the full 21-day dosing schedule (Supplementary Fig. S11).

**Fig. 6:**
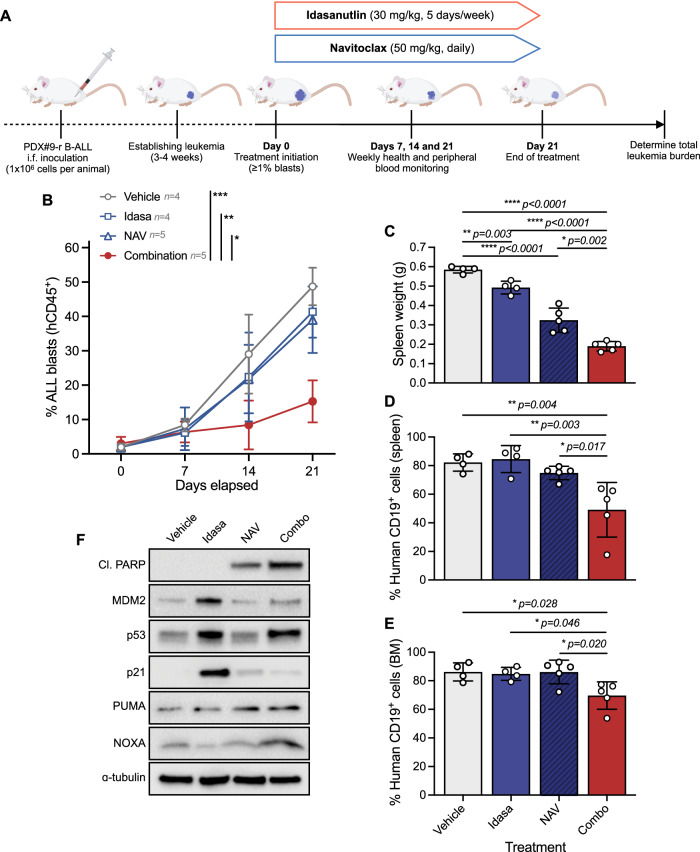
Combination of idasanutlin and navitoclax synergistically inhibits in vivo growth in a high-risk relapsed ALL xenograft model. **A** Schematic of experimental approach. **B** NSG mouse recipients received 1 × 10^6^ viable B-ALL PDX#9-r cells intrafemorally and when human ALL blasts in the peripheral blood reached ≥ 1%, mice were randomized to receive idasanutlin (30 mg/kg) once daily five days per week, navitoclax (50 mg/kg) once daily, and their combination for 21 days by oral gavage. **C** Spleen weights from recipients following treatment with idasanutlin (30 mg/kg) once daily five days per week, navitoclax (50 mg/kg) once daily, and their combination for 21 days by oral gavage. Mice were euthanized within 24 h of their final dose. **D** Quantification of human CD19 + B-ALL cells by flow cytometry analysis of splenocytes from engrafted mice. **E** Quantification of hCD19+ B-ALL cells by flow cytometry analysis of bone marrow from engrafted mice. **F** Steady-state pharmacodynamic analyses of spleen blasts derived from PDX#9-r engrafted mice treated with each treatment arm for three consecutive days (*n* = 3 per group). Immunoblots show induction of cleaved PARP (Asp214), on-target p53 pathway signaling, and enhanced NOXA. ɑ-tubulin served as the loading control. In (**B**–**E**), treatment groups were compared by two-way ANOVA with Tukey post-hoc test and error bars indicate mean±s.d.

To determine the efficacy of the drug combination, we first used a high-risk xenograft model derived from a relapsed B-other (PDX#9-r) patient 6-months post-hematopoietic cell transplant (HCT; Fig. 6A). Following successful engraftment, xenografts were randomized into vehicle control, idasanutlin (30 mg/kg QD on a 5-days-on 2-days-off schedule), navitoclax (50 mg/kg QD), and combination treatment groups. As anticipated, vehicle-treated mice exhibited progressive disease associated with increased leukemia burden over the treatment period (Fig. 6B). Monotherapy idasanutlin or navitoclax achieved very modest, non-significant reductions in peripheral human leukocyte counts (*p* > 0.05). By contrast, combination treatment significantly diminished peripheral leukocyte counts as compared to either monotherapy or vehicle (*p* < 0.011). At the end of the treatment period, spleen weights were significantly decreased in response to idasanutlin (*p* = 0.003) or navitoclax (*p* < 0.0001), but were further decreased in combination treated mice as compared to the respective monotherapies (*p* < 0.002, Fig. 6C). These observations were corroborated by enhanced reductions of human CD19^+^ cells in both spleen (Fig. 6D) and bone marrow (Fig. 6E). Steady-state pharmacodynamic analyses of spleen blasts confirmed on-target drug activity evidenced by enhanced p53 signaling pathway activation in mice treated with idasanutlin as monotherapy or in combination with navitoclax, with combination treated mice additionally showing increased cleaved PARP and NOXA levels (Fig. 6F). Consistent with a preferential engagement of apoptotic cell death over cell cycle arrest, p21 protein levels were significantly diminished in combination-treated mice as compared to idasanutlin-treated mice.

**Fig. 7:**
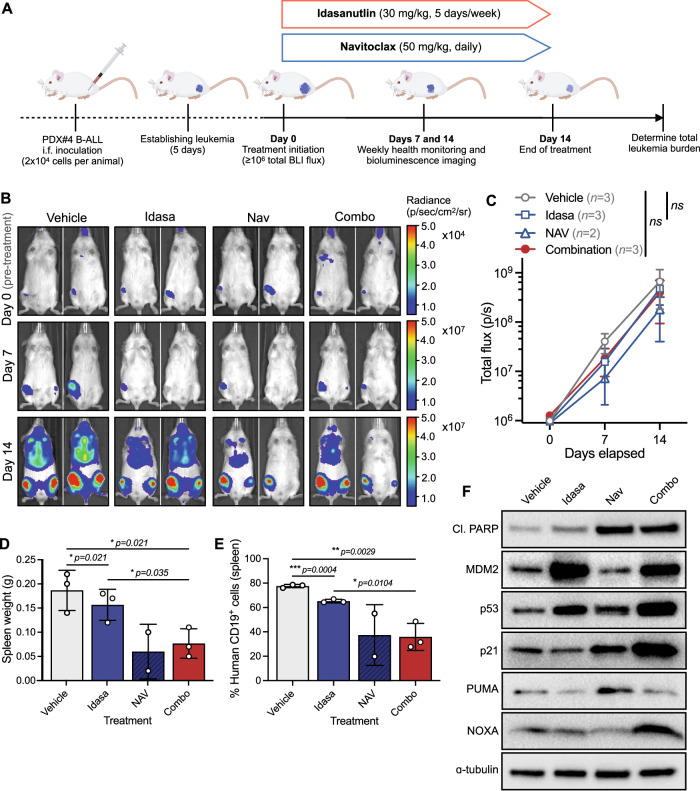
Combination of idasanutlin and navitoclax inhibits in vivo growth of a very high-risk *TCF3::HLF*-rearranged ALL xenograft model. **A** Schematic of experimental approach. **B** NSG mouse recipients received 2 × 10^4^ viable B-ALL PDX#4 cells lentivirally transduced with pUltra-Chili-Luc plasmid to express Firefly luciferase. Engraftment was confirmed by bioluminescence imaging (BLI) 5 days after intrafemoral leukemia cell inoculation. Representative BLI results for days 0 (prior to any treatment), 7, and 14 after start of treatment of recipients treated with idasanutlin (30 mg/kg) once daily five days per week, navitoclax (50 mg/kg) once daily, and their combination for 14 days by oral gavage (*p.o*.). **C** Quantification of BLI by total flux in each treatment group. **D** Spleen weights from recipients four days after treatment with idasanutlin (30 mg/kg) once daily five days per week, navitoclax (50 mg/kg) once daily, and their combination for 14 days by oral gavage (*p.o*.). **E** Quantification of human CD19 + B-ALL cells by flow cytometry analysis of splenocytes from engrafted mice. **F** Steady-state pharmacodynamic analyses of spleen blasts derived from PDX#4 engrafted mice treated with each treatment arm for three consecutive days (*n* = 3 per group). Immunoblots show induction of cleaved PARP (Asp214), on-target p53 pathway signaling, and enhanced NOXA. ɑ-tubulin served as the loading control. In (**C**–**E**), treatment groups were compared by two-way ANOVA with Tukey post-hoc test and error bars indicate mean±s.d.

Finally, we evaluated the therapeutic potential of the drug combination in very high-risk *TCF3::HLF*-rearranged PDX#4 cells lentivirally transduced with a luciferase-expressing vector, allowing for real-time monitoring of disease burden by bioluminescent imaging of luciferase reporter expression [33]. Cell transduction did not substantially alter drug sensitivity in vitro (Supplementary Fig. S12). Following successful engraftment, xenografts were treated with vehicle control, idasanutlin, navitoclax, or combination for 14 days and total body leukemic burden was monitored weekly (Fig. 7A). While there was no significant difference in total body leukemic burden by BLI between drug treatment conditions overall (*p* > 0.05; Fig. 7B, C), at the end of the dosing period there were significant reductions in spleen sizes of combination treated mice as compared to vehicle control or idasanutlin alone (*p* < 0.05; Fig. 7D); confirmed by decreased CD19^+^ leukemic blasts in the spleen of combination-treated mice at the end of treatment (Fig. 7E). Steady-state pharmacodynamic analyses of spleen blasts confirmed on-target drug activity by enhanced p53 signaling pathway activation in mice treated with idasanutlin as monotherapy or in combination with navitoclax, and increased cleaved PARP in drug-exposed spleen blasts (Fig. 7F). Analysis of additional pharmacodynamic markers revealed a strong induction of MCL-1-stabilizing RAS pathway activation in navitoclax-treated mice in vivo, as indicated by enhanced levels of phospho-ERK1/2(T202/Y204) and MCL-1 (Supplementary Fig. S13), which may explain the limited therapeutic response observed in this particular high-risk *TCF3::HLF*-rearranged model.

Collectively, these data confirm that idasanutlin and navitoclax together kill *TP53*-wildtype high-risk and relapsed patient-derived human ALL at subclinical doses in vivo.

## Discussion

Prior studies have demonstrated the feasibility of MDM2 inhibition as a therapeutic strategy in ALL, predominantly focused upon specific subtypes including *KMT2A*-rearranged [34] and *ETV6::RUNX1* translocated ALL [35]. We evaluated the clinically advanced MDM2 inhibitor idasanutlin ex vivo in a comprehensive panel of ALL samples including high-risk subgroups and relapsed disease. Idasanutlin exposure was universally associated with potent, on-target, cytotoxic activity at subclinical concentrations in the absence of *TP53* inactivating mutations, promoting p53 activation as a promising therapeutic strategy in ALL. Amongst the increasing number of MDM2 antagonists entering clinical investigation in recent years [16], idasanutlin has progressed furthest with its use in the phase 3 MIRROS trial (NCT02545283) in combination with cytarabine for the treatment of relapsed or refractory acute myeloid leukemia (AML). Preliminary results from this study demonstrated a promising overall response rate of 38.8% vs 22.0% for cytarabine alone but the trial was subsequently terminated due to poor improvements to overall survival and remission rates [36]. Consistent with other clinical MDM2 antagonists, idasanutlin appears to be well tolerated but the most common adverse events include gastrointestinal toxicities and myelosuppression causing febrile neutropenia and thrombocytopenia. Together with the insufficient clinical responses observed to MDM2 inhibitors as monotherapy [17, 36], this prompts a need to identify optimal drugs for use in combination therapies.

Navitoclax has been associated with clinical activity in early clinical studies across lymphoid malignancies [27, 37]. However, dose-limiting thrombocytopenia, due to on-target BCL-x_L_ inhibition [38], discouraged its further development which was superseded by the selective BCL-2 inhibitor venetoclax [39]. In AML, venetoclax combined with idasanutlin has demonstrated impressive synergistic activity in preclinical in vitro and in vivo studies [32, 40, 41], and shows encouraging efficacy in patients with relapsed or refractory disease [23]. Excitingly, idasanutlin in combination with chemotherapy or venetoclax is currently progressing to phase I/II clinical trials for relapsed pediatric AML and ALL patients (NCT04029688). However, unlike AML blasts which are predominantly BCL-2 dependent [42], ALL blasts often exhibit anti-apoptotic dependence on both BCL-2 and BCL-x_L_ [43–45]; with exception to specific subtypes [44, 46, 47]. Accordingly, Khaw and colleagues showed venetoclax to be effective against only a minority of B-ALL xenografts in vivo, whereas combined BCL-2/BCL-x_L_ inhibition resulted in synergistic killing in most models [47]. Further, venetoclax was not highlighted in our high-throughput screening approach despite being present in the drug library. Recognition of this dual BCL-2/BCL-x_L_ dependence has prompted several ALL trials combining venetoclax with low-dose navitoclax to enhance therapeutic efficacy without the dose-limiting thrombocytopenia associated with navitoclax monotherapy (NCT03181126, NCT05192889). Preliminary efficacy of such a regimen with standard chemotherapy was promising in a heavily pretreated population of relapsed ALL patients, including those with prior HCT or CAR-T therapy [48].

Hotari et al. previously highlighted some anti-proliferative in vitro promise of an idasanutlin-navitoclax combination using three established B-ALL cell lines [49]. More recently, Johansson et al. reported promising in vivo activity of combined idasanutlin and navitoclax in four models of T-ALL, including one relapsed case [50]. This present study is the first to report on the therapeutic efficacy and potential benefit of p53 activation with concurrent BCL-x_L_/BCL-2 inhibition against an extensive range of preclinical models of high-risk and relapsed ALL, demonstrating the broad applicability of the combination in both B- and T-ALL. While both navitoclax and idasanutlin have been associated with thrombocytopenia, we report highly synergistic antileukemic efficacy of the combination in vivo supported by drug plasma concentrations lower than those safely attained in patients [17, 26]; potentially mitigating dose-limiting thrombocytopenia. Crucially, we demonstrate encouraging antileukemic activity of the drug combination at navitoclax plasma concentrations approximately equivalent to “low-dose” navitoclax in contemporary trials [48] and propose a mechanism for its synergistic effects.

Both p53 and BCL-2 family proteins are central regulators of apoptotic machinery [51]. Activation of p53 alone may often be insufficient to engage apoptosis but instead induce cell-cycle regulatory proteins to orchestrate reversible cell cycle arrest, protecting tumor cells from apoptotic cell death and plausibly attests to the limited clinical efficacy of monotherapy MDM2 inhibitors. We show that the idasanutlin-navitoclax combination preferentially engages apoptotic cell death over pro-survival cell cycle arrest in ALL both in vitro and in vivo. We further propose the MCL-1-binding and p53 transcriptionally regulated pro-apoptotic protein NOXA to be implicated in the mechanistic combinatory action. This is of substantial relevance since anti-apoptotic MCL-1 is a well-documented acquired resistance factor towards BCL-2 family inhibitors [52, 53]. Direct therapeutic targeting of MCL-1 exhibits profound toxicity to normal tissues [54], especially cardiac toxicity [55], and so indirect targeting of MCL-1 such as by enhanced NOXA, might represent a viable alternative therapeutic approach. Of note, we did not observe decreased MCL-1 protein expression in response to idasanutlin treatment, in contrast to reported observation in AML [31]. We demonstrate impressive in vitro synergistic activity of the idasanutlin-navitoclax combination across 12/14 primary-derived ALL samples investigated. We further challenged the combination efficacy in vivo using two very high-risk ALL xenografts. In PDX#9-r, derived from a 64-year old patient with relapsed B-other ALL post-HCT, we observed encouraging efficacy of the combination over monotherapies at subclinical plasma concentrations safely achieved in adult patients [17, 48]. The maximum tolerated dose of idasanutlin is still under investigation in pediatric patients (NCT04029688). The *TCF3::HLF*-rearranged PDX#4 xenograft — an almost universally-fatal, rare ALL subtype typically associated with relapse and death within two years from diagnosis [46, 56, 57] — exhibited substantially decreased extramedullary leukemic growth in response to combination therapy but not total leukemia burden at these subclinical plasma concentrations. We further identified activation of the RAS/MAPK signaling axis as a potential mediator of acquired in vivo resistance to the drug combination in this model; a mechanism described previously in studies of venetoclax in AML [53] but not yet reported in ALL.

In summary, our data strongly indicate that combination treatment targeting both MDM2 and BCL-x_L_/BCL2 may provide a novel therapeutic option for patients with relapsed or refractory ALL. These agents are already used independently in clinical trials for other malignancies and we anticipate their rapid introduction into ALL trials.

### Supplementary information

Supplemental material

Supplementary Table S1

Supplementary Table S4

Supplementary Table S6

Supplementary Table S7

## Data Availability

Data generated or analyzed during this study are included in this published article and its supplementary information files, or otherwise available from the corresponding author on reasonable request.
