# Supplementary material for: Combination p53 activation and BCL-xL/BCL-2 inhibition as a therapeutic strategy in high-risk and relapsed acute lymphoblastic leukemia
Source: Leukemia. 2024 Apr 10;38(6):1223–35. doi: 10.1038/s41375-024-02241-7 (PMC11147763; doi:10.1038/s41375-024-02241-7)
Supplement: Supplementary file 1 — Supplemental material [file 41375_2024_2241_MOESM1_ESM.pdf]

Supplementary material for “Combination p53 activation and BCL-x_L/ BCL-2 inhibition as a therapeutic strategy in high-risk and relapsed acute lymphoblastic leukemia”

Hayden L. Bell, et al.

1. SUPPLEMENTARY METHODS
2. SUPPLEMENTARY FIGURES AND LEGENDS
3. SUPPLEMENTARY TABLES AND LEGENDS
4. SUPPLEMENTARY REFERENCES

1. SUPPLEMENTARY MATERIALS AND METHODS

Cell culture

TP53 wildtype B-cell precursor ALL (BCP-ALL) relapsed cell lines NALM6 and RS4;11, were obtained from the American Type Culture Collection (Manassas, VA) and authenticated by STR profiling. *TP53* isogenic NALM6 cells were purchased from Horizon Discovery (Cambridge, UK). hTERT-immortalized mesenchymal stem cells (MSCs) were a gift from D. Campana (National University of Singapore, Singapore). Cell lines were cultured in RPMI-1640 (Sigma-Aldrich, Dorset, UK) with 10% fetal bovine serum (Gibco, Rugby, UK) and 2 mmol/L *L*-glutamine at 37°C in 5% (v/v) CO_2 and were routinely tested for mycoplasma contamination using MycoAlert® (Lonza, Basel, Switzerland). All experiments used genetically authenticated, microbial-free cells in their exponential phases of growth maintained in culture for no longer than 2 months and/or 25 passages.

Compounds

For *in vitro* studies, idasanutlin (RG7388; Adooq Bioscience, CA, USA), navitoclax (ABT-263; Adooq Bioscience), navtemadlin (AMG-232; Cayman Chemicals, MI, USA), and siremadlin (HDM201; MedChem Express, NJ, USA) were dissolved in dimethyl sulfoxide (DMSO). Drug aliquots were stored at -80°C. All negative control groups were treated with DMSO solvent (≤0.2%, Sigma-Aldrich). For *in vivo* studies, idasanutlin and navitoclax were prepared as a suspension in 10% ethanol, 30% PEG 400 (Sigma-Aldrich), and 60% Phosal® 50 PG (Lipoid GmbH, Ludwigshafen, Germany) by ultra-sonication.

In vitro drug treatment and assessment of cytotoxicity in ALL cell lines

For dose-response analysis, 1-3x10^4 ALL cells were plated per well in 100µL culture media in 96-well plates. Cells were incubated at 37°C in 5% (v/v) CO_2 and cell viability/ proliferation analyzed by resazurin after 96 h treatment. For results based on single drug treatment (at least 3 independent experiments), IC_{50} values were determined using a nonlinear regression model in GraphPad Prism 9.

Immunoblotting

Preparation of protein lysates and immunoblotting were performed as previously described (1). Briefly, cells were washed with PBS and lysed in cold RIPA buffer (1% NP-40, 150 mmol/L NaCl, 5 mmol/L EDTA, 0.25% sodium deoxycholate, 50 mmol/L Tris-HCL pH 7.5, 0.1% SDS) supplemented with protease (cat. no. 5892791001, Roche, Hertfordshire, UK) and phosphatase inhibitors (cat. no. 4906845001, Roche). Proteins were separated using

4-20% gradient SDS-polyacrylamide gels (Bio-Rad Laboratories, Hercules, CA), then electrophoretically transferred onto polyvinylidene fluoride membranes. Membranes were blocked with 5% (w/v) skimmed milk or 5% (w/v) BSA in TBST for 1h, and then incubated with primary antibodies outlined in Supplementary Table S2 in blocking buffer overnight at 4°C. Secondary anti-rabbit or anti-mouse IgG HRP-conjugated antibodies (Agilent, Stockport, UK) were used. Signals were visualized using chemiluminescence (GE Healthcare, Chicago, IL) on a Bio-Rad Gel Doc XR System. Densitometry was performed with ImageJ v2.1.

Drug screening

The DiscoveryProbe FDA-approved drug library of 1971 drugs was purchased from APExBIO technology (Houston, TX) and annotated using data from the BROAD Institute Drug Repurposing Hub (2) and the NCI Inxight (3) databases. The full drug list is available in Supplementary Table S4. To minimize compound instability and potential batch effects between screens arising from freeze-thaw cycles, 1 mM compound plates were prepared in DMSO from master stocks using a Biomek FXp liquid handler (Beckman Coulter, Brea, CA) in a sterile cabinet. Plates were stored at -20°C and underwent ≤ 3 freeze-thaw cycles.

For primary screening, the drug library was screened in quadruplicate technical replicates using the NALM6 cell line for three independent replicates. Drugs were constituted in RPMI-1640 (+10% FBS) medium and achieved 1 μM final concentration (0.1% DMSO). Cells were dispensed into compound-containing black 384-well plates (Greiner Bio-One, Stonehouse, UK) previously loaded using a Biomek FXp liquid handler, such that each well contained $5x10^3$ cells. For drug combination screening, idasanutlin (30 nM final concentration) or control vehicle (DMSO) were added to cell suspensions immediately preceding cell seeding (≤0.2% final DMSO). Plates were incubated for 96 hours in a humidified chamber 37°C, 5% CO_2. After incubation, 10X resazurin was added to each well using a Biomek FXp liquid handler; plates were immediately protected from light and stored at 37°C, 5% CO_2 until data acquisition. Quality control metrics were calculated using a Python script available at https://github.com/hayden-bell/HTS-QC. Positional effects were determined using iterative row-by-row Welsh t-tests (p=0.05 threshold) implemented in Python using the scipy.stats package and corrected by LOESS normalization. Putative hits were subjected to five-point log dose ranges (0.1 nM to 1 μM) of each drug in technical quadruplicate to estimate IC_{50}s of each compound ± idasanutlin IC_{25} (30 nM) in the NALM6 cell line (n=3).

B- (n=5) and T-ALL (n=1) patient-derived xenograft (PDX) samples were subjected to five-point log dose ranges (0.1nM - 1 µM) of each of the top-ranked FDA-approved drug hits (n=32) ± idasanutlin (respective PDX IC_{40} and IC_{60}s) in co-culture with hTERT-immortalized MSCs. Mean cell survival was determined in technical triplicate after 96 hour drug exposure using automated image-based microscopy as described (4) and analyzed by four-parameter logistic regression using the scipy.stats and scipy.optimize python packages.

DNA and RNA isolation

Genomic DNA was extracted from mononuclear preparations of primary or PDX specimens using a QIAamp DNA Mini Kit (#51304, QIAGEN, UK) according to manufacturer's protocol. For RT-qPCR analysis, total RNA was extracted from leukemic cells using the RNeasy Mini Kit (QIAGEN, UK) according to manufacturer's protocol. The concentration and quality of isolated nucleic acid were determined by spectrophotometry (NanoDrop ND-1000, Thermo Scientific, UK).

Targeted *TP53* exon mutation sequencing

Primers for *TP53* exons 4 to 8 were used to amplify genomic DNA by PCR using an AmpliTaq Gold DNA Polymerase DNA kit (#4311806, ThermoFisher Scientific). Primer sequences and thermal cycling conditions for individual amplicons are shown in Supplementary Table S9. Following agarose gel electrophoresis to verify amplicon size and PCR clean-up (QIAquick PCR purification kit, #28104, Qiagen), amplicons were outsourced to Source Bioscience (Cambridge, UK) for Sanger sequencing using both forward and reverse primers. The analysis of chromatograms and alignment with human *TP53* NCBI reference sequence (NM_000546.6) was conducted with Snap Gene v6.2 Software (Boston, MA).

RT-qPCR

2 µg RNA was used for cDNA synthesis using the High-Capacity cDNA Reverse Transcription kit (ThermoFisher Scientific). The expression of target gene *PMAIP1* and endogenous control gene *TBP* was measured in technical triplicate by a fluorescence-based kinetic RT-qPCR using the following TaqMan™ Gene Expression Assays: Hs00560402_m1, Hs99999910_m1 (Applied Biosystems) and TaqMan™ Gene Expression Master Mix (Applied Biosystems). The reaction was performed using a ViiA7 Real-time PCR System (Applied Biosystems) in accordance with the manufacturer's instructions and data processing was performed using QuantStudio Real Time PCR software (v1.3). For

data analysis, the relative gene expression (fold-change) relative to respective control was calculated using cycle threshold (Ct) values with the formula: fold-change = $2^{-\Delta Ct}$; ΔCt = Ct(target) - Ct(reference).

siRNA-mediated knockdown of NOXA

Approximately 1×10^7 cells were resuspended in opti-MEM reduced serum medium (ThermoFisher Scientific) and subjected to electroporation with a BTX Gemini X2 electroporation system (BTX, Holliston, MA, USA) using 4 mm gap cuvettes under the following conditions: 350V, 10 ms, 1 pulse, square wave. Knockdown experiments used ON-TARGETplus Human *PMAIP1* SMARTPool siRNA (Dharmacon, Lafayette, CO, USA) or ON-TARGETplus (Dharmacon) non-targeting pool as a negative control; target sequences are provided in Supplementary Table S10. Following electroporation, cells were cultured in standard medium at a density of 1×10^6 cells/mL. Following 24 hours transfection, expression of target gene products was assessed using immunoblotting and cells were subjected to subsequent analyses.

Determination of *in vivo* plasma drug concentrations

Plasma drug concentrations of idasanutlin and navitoclax were evaluated in NSG mice after a 30 mg/kg idasanutlin (*n*=3), 50 mg/kg navitoclax (*n*=3), or both (*n*=3) oral dose (10% ethanol / 30% PEG 400 / 60% Phosal® 50 PG) by oral gavage. Sample size was calculated by resource equation approach as described in ref. (5). The steady state plasma concentrations of both drugs alone and in combination were examined on the third day of three consecutive days of daily dosing. At 3 hours after drug administration, mice were exsanguinated by cardiac puncture under isoflurane inhalation. Plasma was isolated from heparinized blood samples by centrifugation (14 000 rpm x 5 minutes, 4°C) and immediately frozen. Analysis of plasma concentrations for both drugs was carried out by liquid chromatography-mass spectrometry (LC-MS/MS) analysis and calibrated using standards freshly prepared in drug naïve NSG mouse plasma. Chromatographic separation of idasanutlin was achieved using a 1260 series Infinity high-performance liquid chromatography system (Agilent, Cheshire, UK) coupled with an Agilent 6460 triple quad mass spectrometer. For separation and quantification of navitoclax a Prominence high-performance liquid chromatography system was coupled with an AB Sciex API 4000 triple quadruple liquid chromatography-mass spectrometer (Applied Biosystems, CA, USA). Both assays used a Zorbax Eclipse Plus 1.8 µm C18 column (50mm x 2.1mm) for peak separation. Aliquots of 20 µL of plasma were analyzed for both drugs and data acquisition and processing were carried out using MassHunter software.

***In vivo* toxicity study of idasanutlin+NAV combination**

Male (*n*=2) and female (*n*=3) 8-12 weeks old NSG mice were treated with idasanutlin (30 mg/kg, once daily 5-days-on 2-days-off) and navitoclax (50 mg/kg, once daily) by oral gavage in 10% ethanol, 30% PEG 400, and 60% Phosal® 50 PG for 21 days. Mouse health was monitored daily as described below. Sample size was calculated by resource equation approach as described in ref. (5) and investigators were not blinded to allocation during experiments and outcome assessment.

Conversion of murine doses to human equivalent doses

Human equivalent doses were calculated according to FDA guidelines and ref. (6), using a mouse to human conversion constant K_m = 0.081 and human body weight of 60 kg. Human equivalent doses were calculated to 2.4 mg/kg for idasanutlin and 4.1 mg/kg for navitoclax.

***In vivo* efficacy studies of idasanutlin-navitoclax antileukemic activity**

Cryopreserved BCP-ALL PDXs were thawed and injected intrafemorally into male and female 8-12 weeks old NSG mice under isoflurane inhalation anesthesia with subsequent analgesia, with $2x10^4$ to $1x10^6$ cells each. Leukemic engraftment was monitored weekly in the murine peripheral blood from tail vein venepuncture as described. The percentage of human cells was presented as ($hCD45^+/mCD45^+$) x 100%. For PDX#4, blasts were lentivirally transduced to stably express dTomato and firefly Luciferase using a pUltra-Chili-Luc vector (AddGene #48688; as previously described ref. (7)) and engraftment was monitored by bioluminescent imaging (BLI) following intraperitoneal injection of 150 mg/kg luciferin (Promega, Southampton, UK) using an IVIS Spectrum imager (Caliper Life Sciences, MA, USA). BLI data were visualized and quantified using LivingImage software (Perkin-Elmer). Leukemic cells at >1% in the peripheral blood or >10^6 p/s total BLI flux in all mice was the starting point for drug administration. Randomized mice (*n*=5 per group or *n*=4 for PDX#4) were treated with 30 mg/kg of idasanutlin (by oral gavage, once daily 5-days-on 2-days-off), 50 mg/kg of navitoclax (once daily), combination of both drugs, or vehicle control. Sample size was calculated by resource equation approach as described in ref. (5). One animal (PDX#4) experienced procedural death after one drug treatment dose and was excluded from analysis. Leukemic burden was determined once per week by peripheral blood monitoring or total body bioluminescence. Pre-determined objective end-points were >10^9 p/s total IVIS flux, >50% blasts in the peripheral blood, or at the end of the dosing regimen at which point animals were humanely killed; preceding appearance of clinical signs of ill heath and avoidance of animals *in extremis.* At necropsy, bone

marrow cells were harvested from tibiae, femurs, and iliac crests by bone crushing and the spleen was minced through a cell strainer before engraftment analysis by flow cytometry. For steady-state pharmacodynamic studies, spleen cells treated for 51 h (3 h after receiving the third daily drug dose) were harvested by mechanical disruption and cryopreserved cells (>80% leukemic blasts) were processed as described. Animals were randomized into treatment groups using a computer based random order generator and investigators were not blinded to allocation during experiments and outcome assessment.

General humane end-points for *in vivo* studies

Mice were observed daily by trained technicians and clinically assessed, using non-aversive handling methods, at least twice weekly to ensure good health. All procedures and handling were performed aseptically. Mice were humanely killed by cervical dislocation at the end of each study or if they displayed any of the following signs: >10% weight loss for 3 days or >15% weight loss from their highest previous weight; abdominal distention, hind limb paralysis, pale extremities, abnormal behavior, or irregular breathing patterns.

2. SUPPLEMENTARY FIGURES AND LEGENDS

Supplementary Figure S1

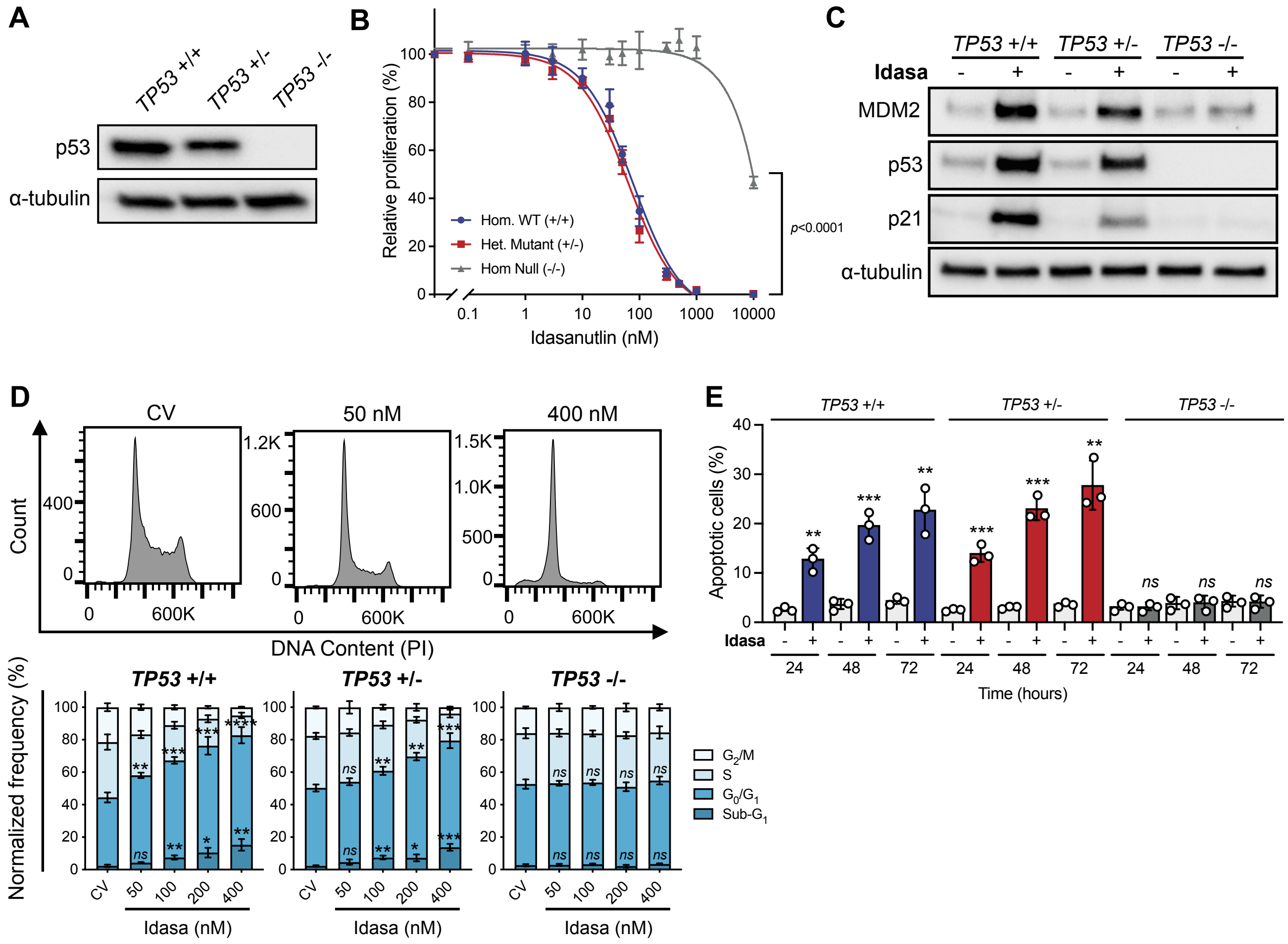

Supplementary Figure S1. MDM2 inhibitor idasanutlin activates p53 signaling, induces cell-cycle arrest, and promotes apoptosis in a p53-dependent manner. A Immunoblot showing p53 expression in *TP53* wildtype cells and stable *TP53* knockout in homozygous null NALM6 cells. α-tubulin was used as a loading control. **B** Isogenic NALM6 cells were exposed to indicated concentrations of idasanutlin for 96 h and subjected to resazurin proliferation assay. The viable cells are presented as a percentage of vehicle-treated cells (control). Dose-response curves were compared using two-way ANOVA. **C** The isogenic NALM6 cells were exposed to respective IC_{50}s of idasanutlin (or 1 μM for $p53^{null}$) for 6 h and protein levels of MDM2, p53, and p21 were detected by immunoblotting. **D** Cell cycle analysis of *TP53* isogenic NALM6 cells exposed to indicated idasanutlin concentrations for 24 h. Representative cell cycle profiles of parental NALM6 cells are shown. Idasanutlin exposed cells were compared to respective control vehicle (CV) using unpaired *t*-test. **E** Exposure of phosphatidylserine (annexin V) was analyzed by flow cytometry in the isogenic NALM6 cells exposed to respective IC_{50}s of idasanutlin (or 1 μM for $p53^{null}$) for indicated times. Idasanutlin exposed cells were compared to respective CV using unpaired *t*-test. All experiments were performed in *N*=3 independent experiments and error bars indicate mean ± SD. *ns=not significant, *p* value < 0.05, ***p* value < 0.01, ****p* value < 0.001, *****p* value < 0.0001.

Supplementary Figure S2

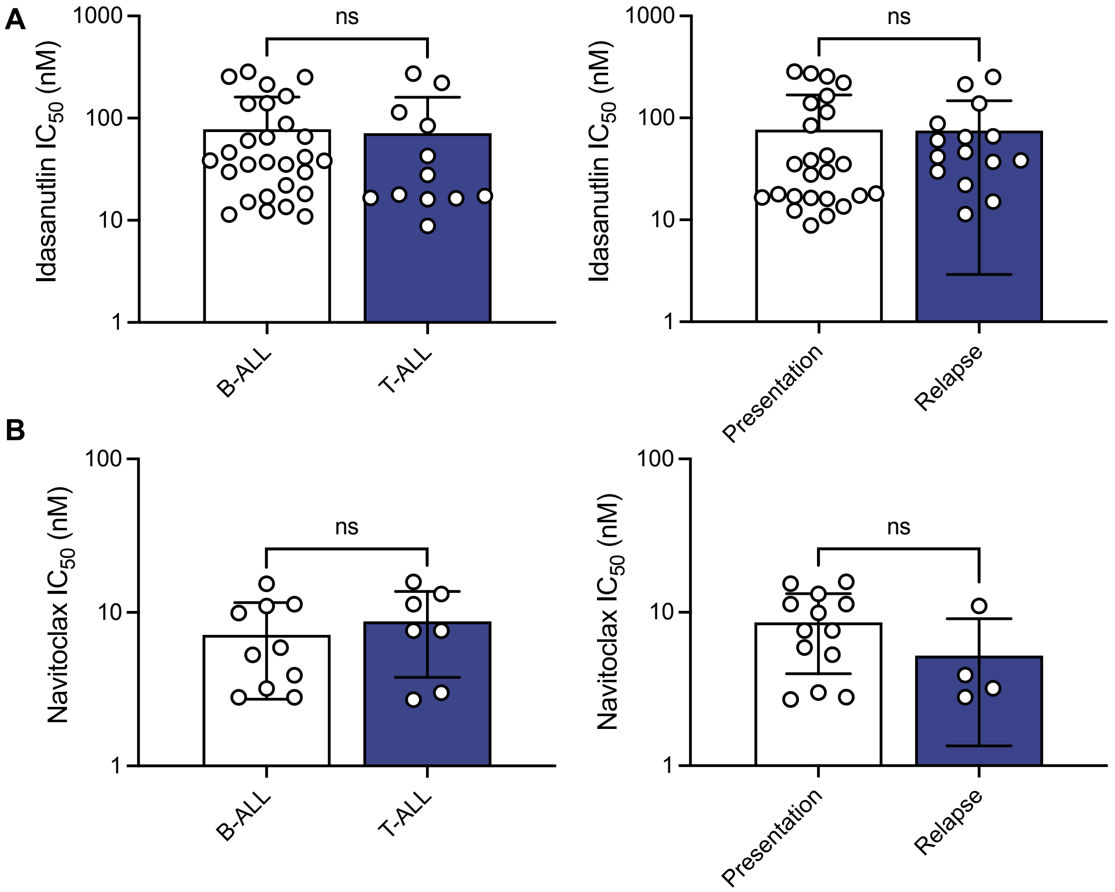

Supplementary Figure S2 *Ex vivo* ALL sensitivity to idasanutlin or navitoclax is not associated with lineage- or disease-status differences. IC_{50} drug sensitivity values of B- and T-lineage ALL samples exposed to **A** idasanutlin or **B** navitoclax were stratified by lineage and/or presentation/relapse status and compared by Welsh *t*-test. Error bars show mean ± standard deviation. *ns*, not significant.

Supplementary Figure S3

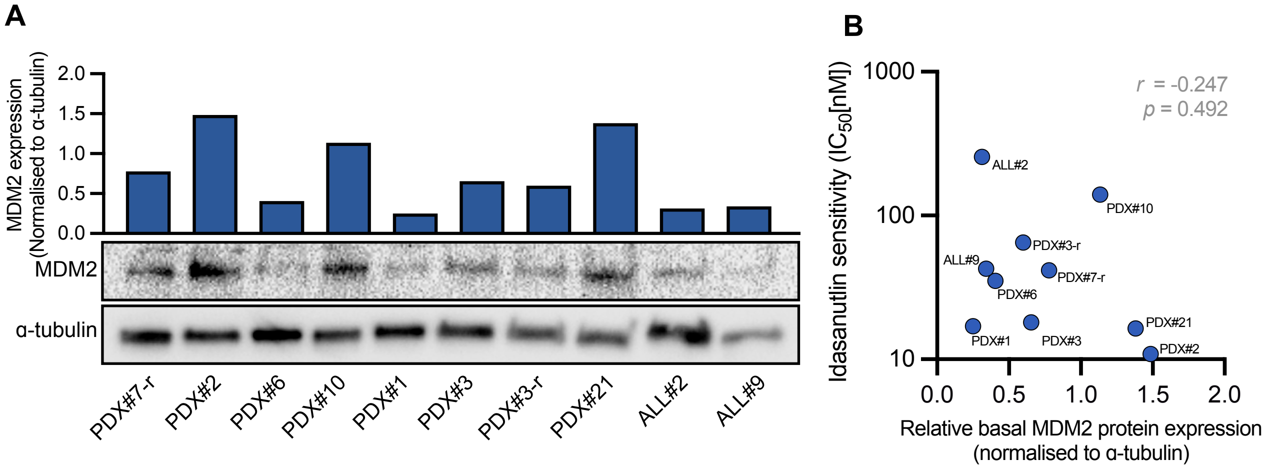

Supplementary Figure S3 ALL blast basal MDM2 protein expression does not significantly correlate with *in vitro* sensitivity to idasanutlin. A Basal MDM2 protein expression in primary (*n*=2) and PDX (*n*=8) ALL blasts was determined by immunoblotting. α-tubulin served as the loading control. Densitometric analysis was performed to quantify relative MDM2 protein expression (normalized to α-tubulin control) for each sample. *N*=1 **B** Scatter plot comparing *in vitro* idasanutlin sensitivity with respective basal MDM2 protein expression. Pearson correlation; *r,* correlation coefficient; *p*, significance.

Supplementary Figure S4

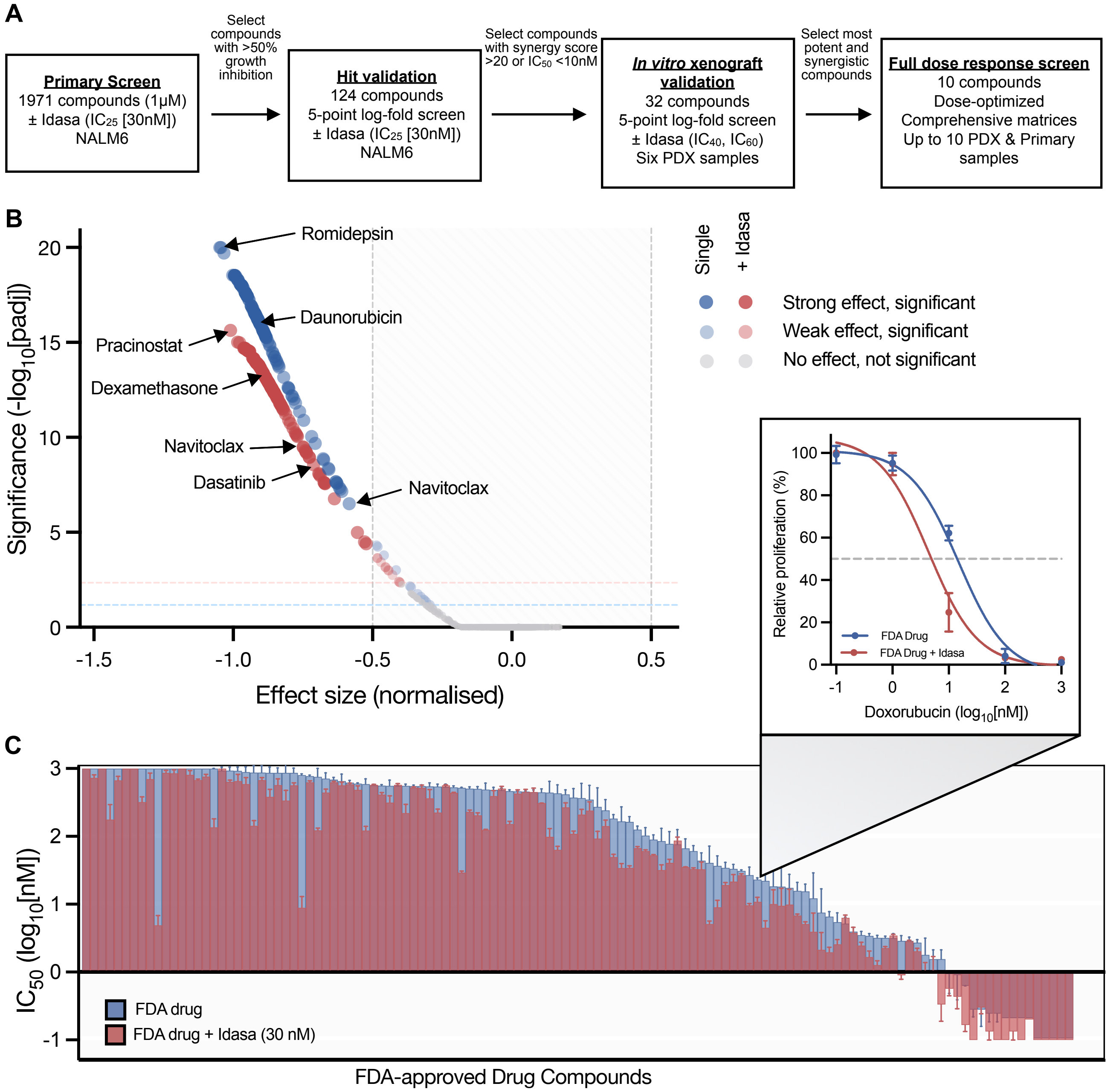

A Strategy for combination drug screening to identify candidate drug partners **B** Volcano plot of drug activity when NALM6 cells were exposed to 1 μM of each screening library drug ± idasanutlin (IC_{25} [30 nM]) for 96 h determined by resazurin-based viability assay (blue points = single-agent library drug exposure, red points = library drug exposure with idasanutlin). *N*=3 independent experiments in quadruplicate. Data were normalized using LOESS regression and expressed as mean normalized Z-score effect. *P* values were adjusted for multiple comparisons by two-stage Benjamini-Hochberg, Krieger, and Yekutieli False Discovery Rate (FDR) procedure. Blue and red colored dashed horizontal lines correspond to FDR=0.01 for single drug and drug+idasanutlin, respectively; drugs below these lines are labeled “not significant”. The two dashed vertical lines correspond to a change of ±0.5 in activity from the dataset and drugs outside this range are labeled “strong effect”. Drugs with a significant but weak effect are labeled as such. Primary screening data are provided in Supplementary Table S4. Quality control data are provided in

Supplementary Table S5. **C** All drugs from primary screening which yielded a significant strong effect were screened at a 5-point $\log_{10}$-dose range (0.1 nM to 1 μM) ± idasanutlin (30 nM) to determine respective IC_{50}s. Data were normalized to vehicle control or idasanutlin-treated cells to examine drug library effects specifically. Estimated IC_{50}s below 0.1 nM were bounded to the minimum tested concentration. Inset shows example data from the doxorubicin-idasanutlin dose-response curves. *N*=3 independent experiments in quadruplicate. Error bars show mean±s.e.m. Drug sensitivity data are provided in Supplementary Table S6.

Supplementary Figure S5

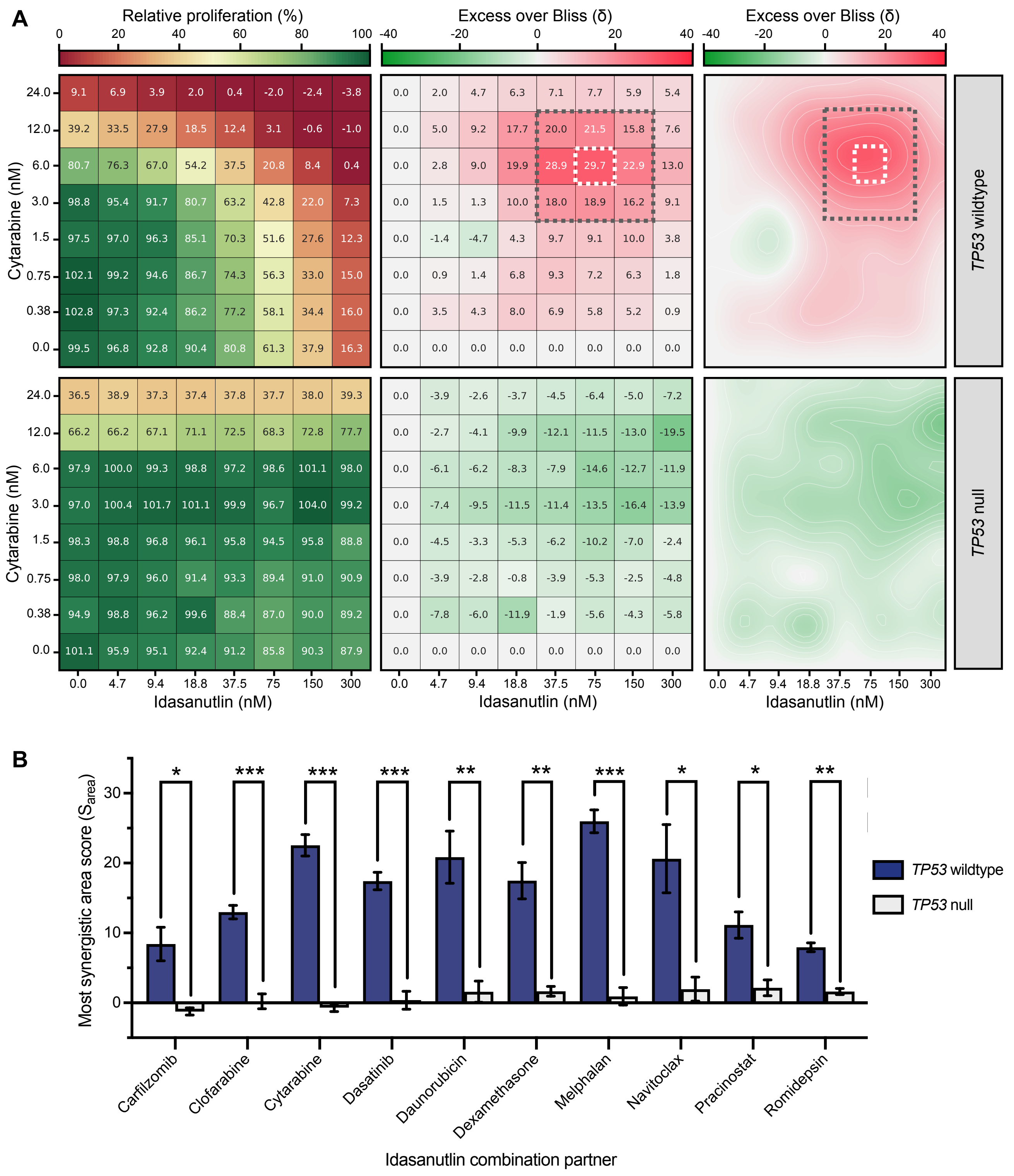

Supplementary Figure S5 Counter screening candidate FDA-approved compounds in a *TP53* isogenic relapsed B-ALL cell line confirms p53-dependent combinational activity. A Representative pairwise combination of idasanutlin with cytarabine in p53^{wt} (top) and p53^{null} (bottom) *TP53* isogenic NALM6. Cells were subjected to increasing doses of idasanutlin or cytarabine alone, or a combination of the two simultaneously for 96 h, and subjected to resazurin proliferation assay. Heat map (left) represents mean viability relative to vehicle-only control. Excess over Bliss synergy scores (center) were calculated at each dose combination (<0, antagonism; 0,

additive; >0 synergy). Two-dimensional contour plots (right) represent the drug interaction landscape for the indicated combination concentrations derived from spline interpolation of the synergy scores. Dark grey dashed box indicates the most synergistic area with the S_{area} derived from the mean of these values. White dashed box indicates synergy max (S_{max}). **B** S_{area} for each candidate RG combination partner pair in the $p53^{wt}$ and $p53^{null}$ isogenic cell lines. *N*=3 independent experiments and error bars indicate mean ± s.e.m. Unpaired *t*-tests, *p value < 0.05, **p value < 0.01, ***p value < 0.001

Supplementary Figure S6

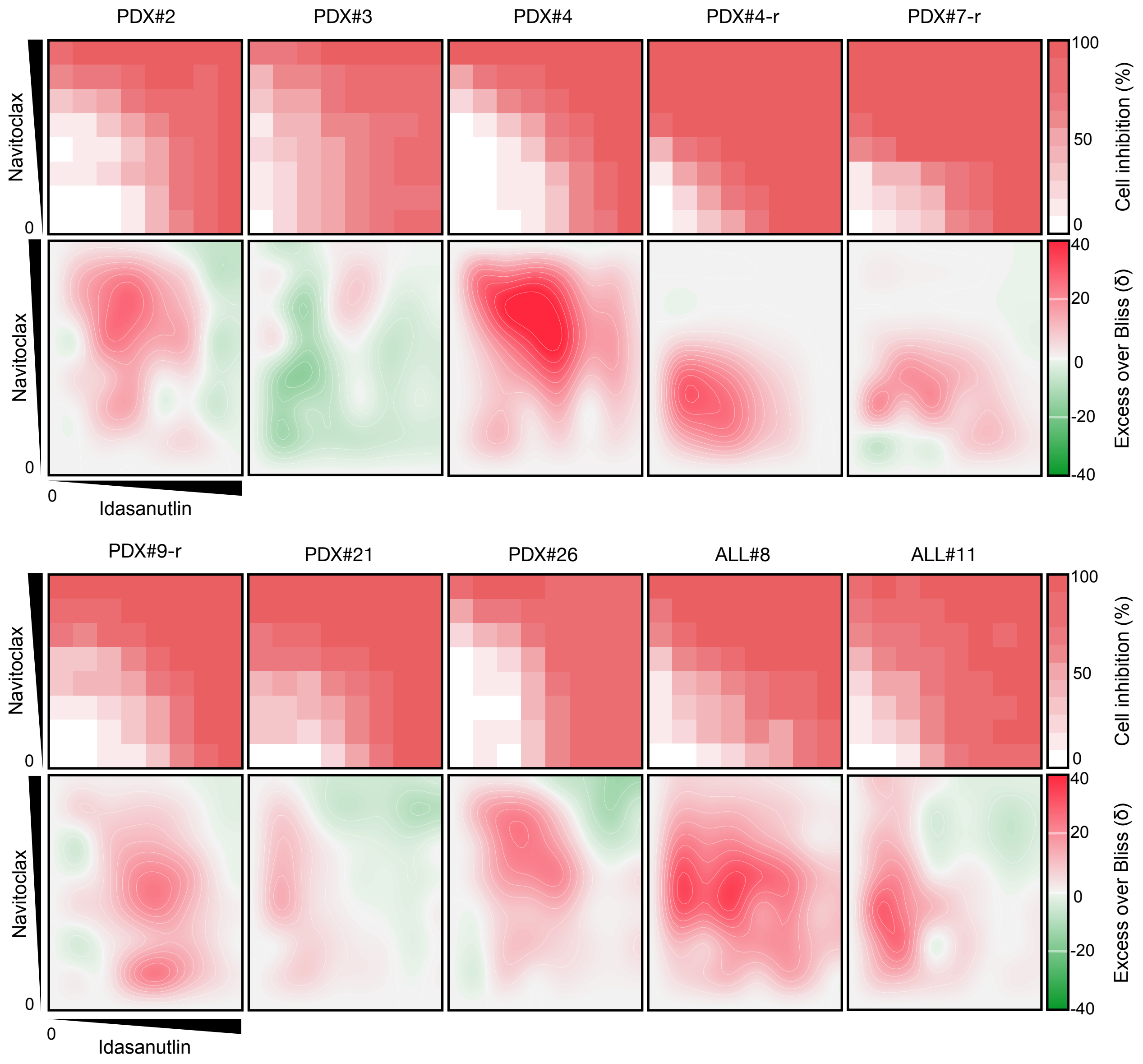

Supplementary Figure S6. Navitoclax significantly augments idasanutlin anti-leukemic activity across a broad range of dose-combination ratios in high-risk primary and PDX ALL samples. Dose-response matrix analyses showing cell inhibition and synergistic landscape across diverse idasanutlin-navitoclax (NAV) dose combinations in additional PDX (*n*=8) and primary ALL (*n*=2) blasts derived from patients with ALL of diverse subtypes. Blasts were subjected to increasing doses of idasanutlin or NAV alone, or a combination of the two simultaneously for 96 h in *ex vivo* coculture with hTERT-immortalized MSCs, and live cell numbers were enumerated by image-based microscopy with post-acquisition image analysis. *N=1* independent experiments in at least technical duplicate.

Supplementary Figure S7

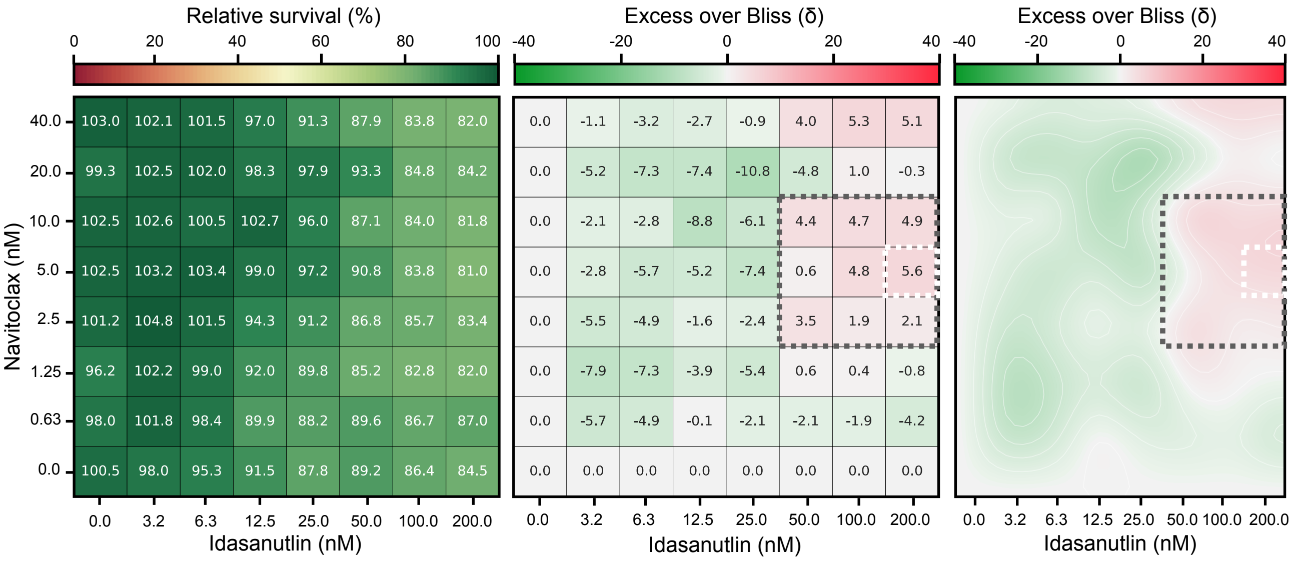

Supplementary Figure S7. Navitoclax does not augment the activity of idasanutlin in bone marrow-derived MSCs at concentrations effective in ALL blasts. The effect of idasanutlin with navitoclax was investigated in hTERT-immortalized MSCs co-cultured with different PDX ALL cells *in vitro.* Cells were subjected to increasing doses of either drug alone, or a combination of the two simultaneously for 96 h, and live cell numbers were determined by image-based microscopy with post-acquisition image analysis. The viable cells are presented as a percentage of vehicle-treated cells (control). Heat map (left) represents mean viability relative to vehicle control. Excess over Bliss synergy scores (center) were calculated at each dose combination (<0, antagonism; 0, additive; >0 synergy). Two-dimensional contour plots (right) represent the drug interaction landscape for the indicated combination concentrations derived from spline interpolation of the synergy scores. Dark grey dashed box indicates the most synergistic area with the S_{area} derived from the mean of these values. White dashed box indicates synergy max (S_{max}). *N*=3 independent experiments in at least technical duplicate with alternative PDX samples.

Supplementary Figure S8

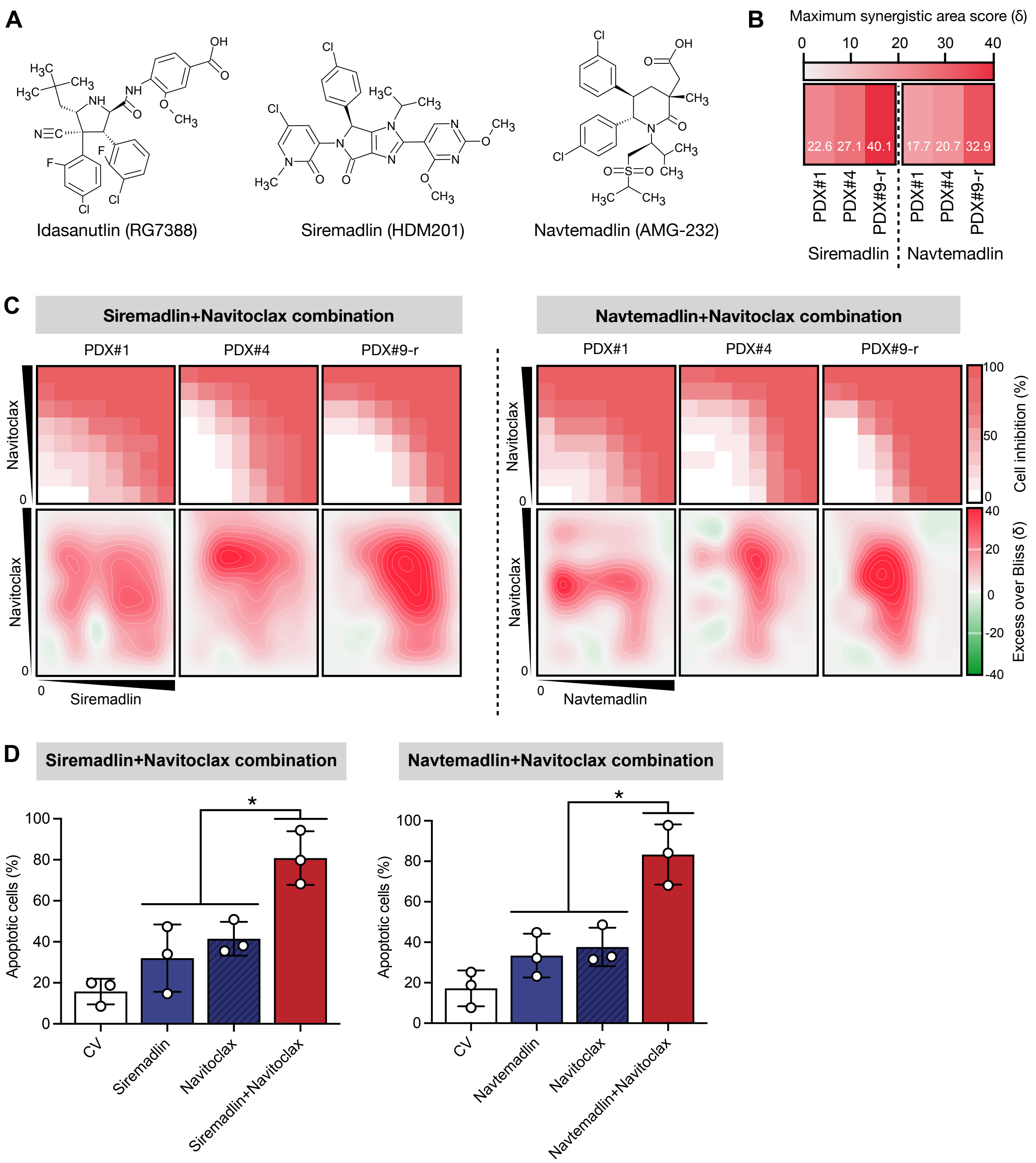

Supplementary Figure S8. p53 activation by structurally-diverse p53-MDM2 binding antagonists recapitulates synergistic interaction with BCL-x$_L$/BCL-2 inhibition by NAV in ALL blasts. A Structure of investigated p53-MDM2 binding antagonists. Available structures were obtained from PubChem database (8) with accession numbers CID 53358942 (idasanutlin); CID 71678098 (siremadlin); CID 58573469 (navtemadlin) and plotted by ACD/ChemSketch, V2021.1. **B** Heat map of most synergistic area Bliss synergy scores (S_{area}) for siremadlin with NAV (left) or navtemadlin with NAV (right) combination activity in PDX (*n*=3) ALL samples in *ex vivo* co-culture with hTERT-immortalized MSCs. *N*=1 independent experiments in duplicate. **C** Dose-response matrix analyses showing cell inhibition (top) and synergistic landscape (bottom) across diverse

siremadlin-NAV (left) or navtemadlin-NAV (right) dose combinations. **D** Apoptotic cells were quantified by annexin-V flow cytometry in PDX ALL blasts (*n*=3) following exposure to respective IC_{50} concentrations of siremadlin or navtemadlin, NAV, and their respective combinations. Error bars show mean ± SD. Combination treated cells were compared to respective single drugs by paired *t*-tests, $^{**}p$ value < 0.01.

Supplementary Figure S9

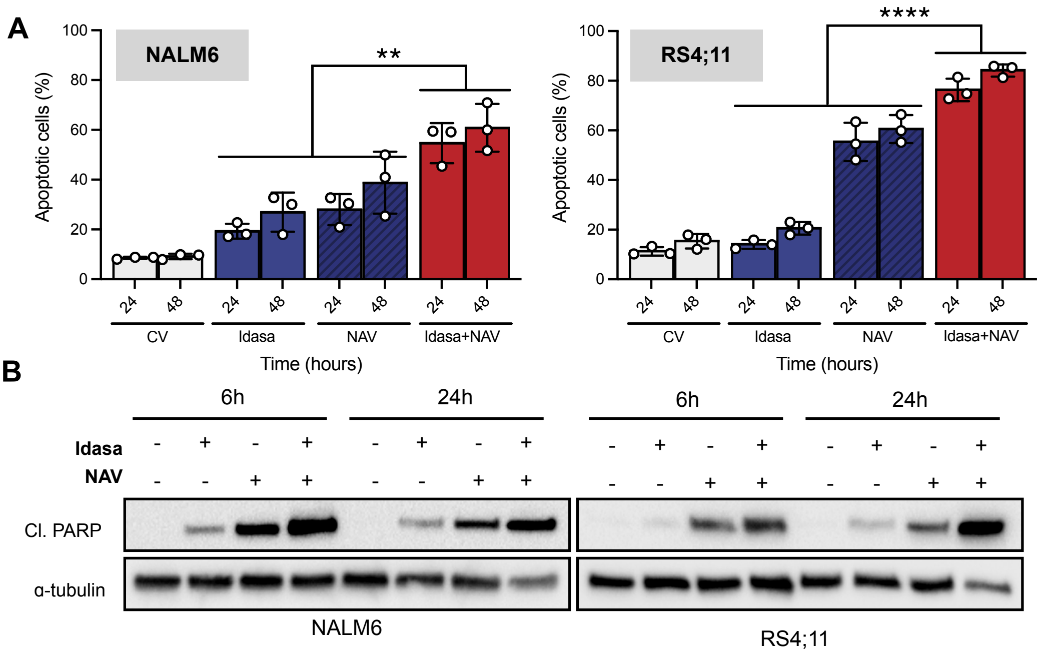

Supplementary Figure S9. p53 activation by idasanutlin synergistically enhances apoptotic cell death with navitoclax in relapsed B-ALL cell lines NALM6 and RS4;11 *in vitro.* A Apoptotic cells were quantified by annexin-V flow cytometry in NALM6 (left) and RS4;11 (right) cells following exposure to respective IC_{50} concentrations of idasanutlin, navitoclax, or their combination for 24 and 48 h. Error bars show mean ± SD of three independent experiments. Combination treated cells were compared to respective single drugs by paired *t*-tests, *****p* value <0.0001, ***p* value < 0.01. **B** Immunoblots showing induction of cleaved PARP (Asp214)(*N*=3 independent experiments) in NALM6 (left) or RS4;11 (right) cells following exposure to respective IC_{50} concentrations of single drugs or their combination for 6 and 24 h. α-tubulin served as the loading control.

Supplementary Figure S10

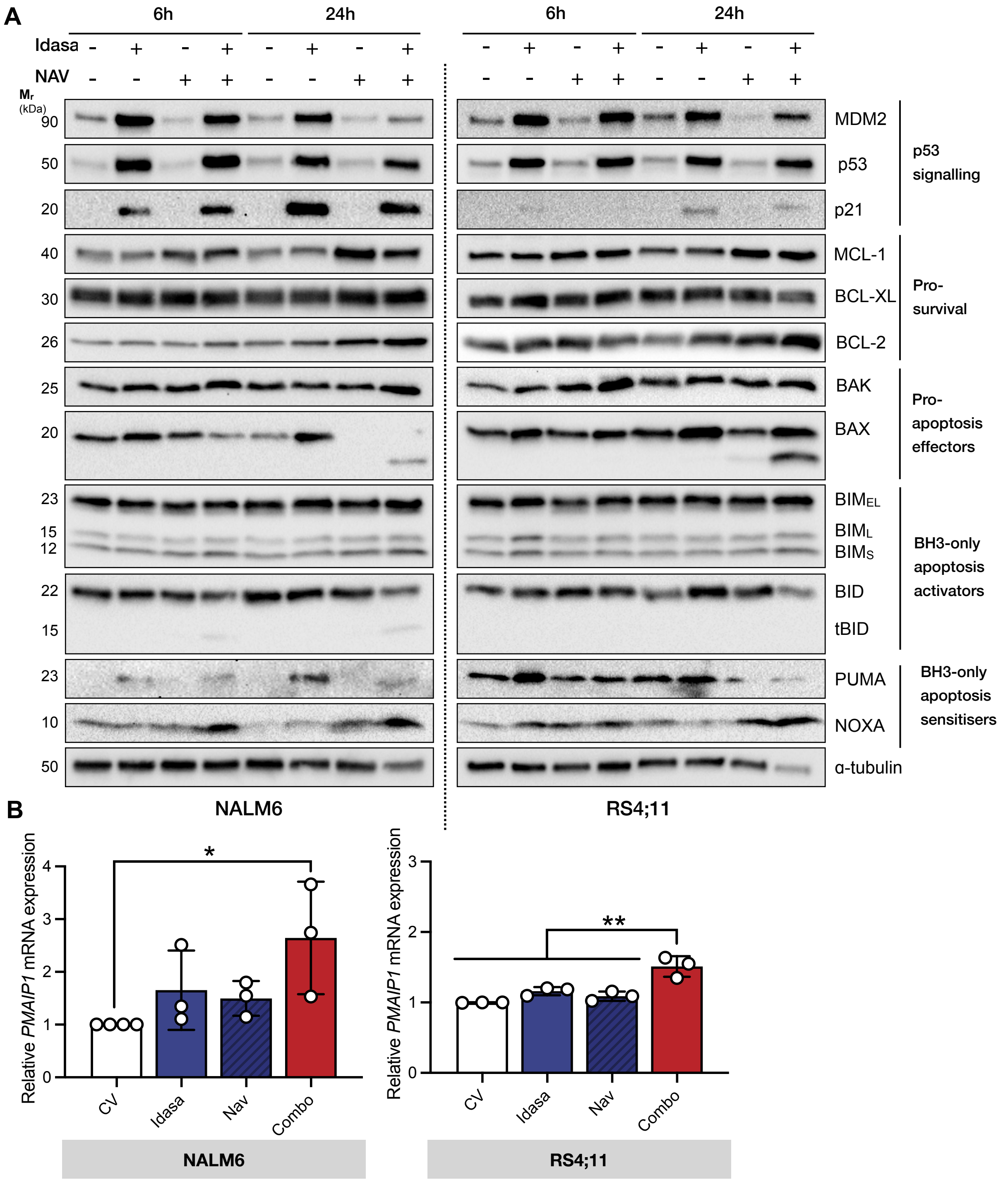

Supplementary Figure S10. Idasanutlin and navitoclax combination treatment strongly up regulates pro-apoptotic protein NOXA in relapsed BCP-ALL cell lines NALM6 and RS4;11. A NALM6 and RS4;11 cells were exposed to respective IC_{50} concentrations of idasanutlin, NAV, both, or control vehicle for 6 and 24 hours. Immunoblot analysis was performed for: p53 pathway proteins p53, MDM2, and p21; BCL-2 family pro-survival proteins MCL-1, BCL-2, and BCL-xL; BCL-2 family pro-apoptotic effector proteins BAK and BAX; and BCL-2 family pro-apoptotic BH3-only proteins BIM, BID, PUMA, and NOXA. BAX immunoblots show full length 21 kDa BAX and truncated 18 kDa BAX which may exhibit enhanced apoptogenic properties (9, 10). Alpha-tubulin served as a loading control. Images are representative of three independent experiments with

exception to PUMA (*n*=1). **b** mRNA expression levels of *PMAIP1* were determined in NALM6 and RS4;11 cell lines following exposure to respective IC_{50} concentrations of idasanutlin, NAV, both, or control vehicle for 24 hours. Data were normalized to internal housekeeper *TBD.*

Supplementary Figure S11

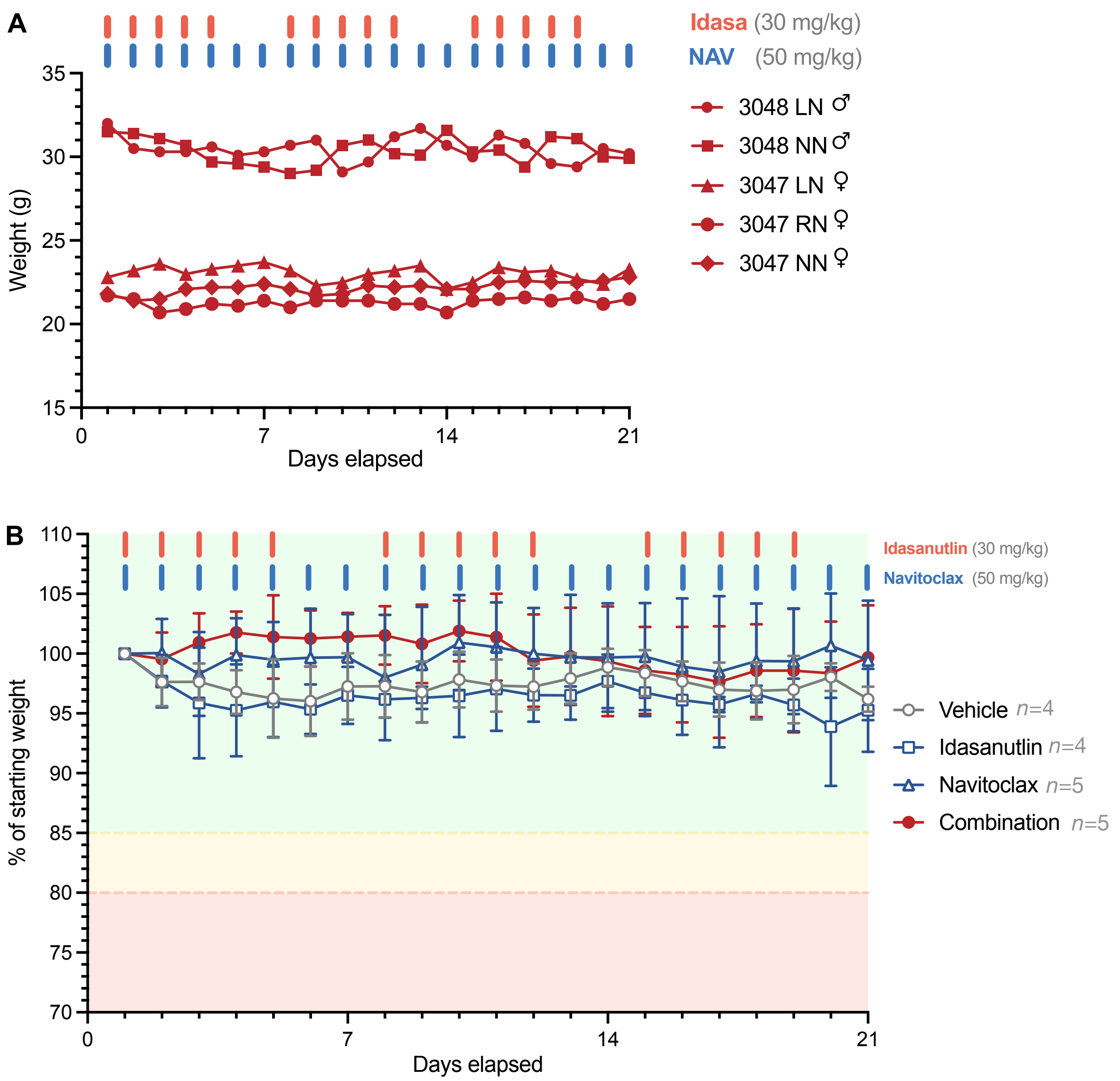

Supplementary Figure S11. Toxicity study of idasanutlin and navitoclax combination *in vivo.* **A** Absolute body weight changes of NSG male (*n*=2) and female (*n*=3) mice in a preliminary toxicity study to determine the safety and tolerability of combination dosing of 30 mg/kg idasanutlin (5 days per week) and 50 mg/kg NAV (daily) by oral gavage (*p.o.*). **B** Retrospective relative weight changes of male and female NSG mice (*n*=4-5 per group) to each drug treatment group with concomitant leukemia burden.

Supplementary Figure S12

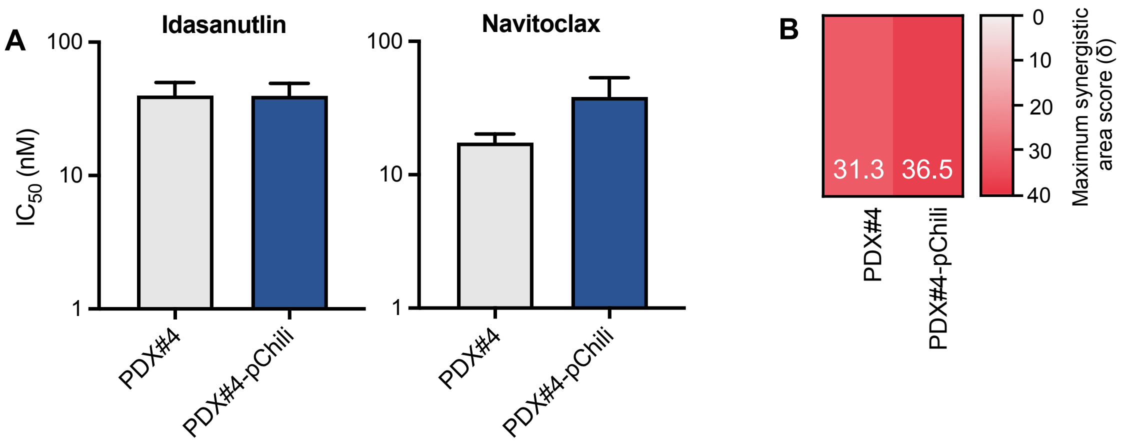

Supplementary Figure S12. Lentiviral transduction of PDX#4 using a pUltra-Chili-Luc plasmid did not substantially affect *in vitro* drug sensitivity. A *In vitro* IC_{50} drug sensitivity values of untransduced and transduced PDX#4 cells exposure to respective single drugs for 96 hours in *in vitro* co-culture with hTERT-immortalized MSCs. *N*=2 independent experiments in technical triplicate. **B** Heat map of most synergistic area Bliss synergy scores for the idasanutlin-navitoclax combination *in vitro. N*=1 independent experiment in technical triplicate.

Supplementary Figure S13

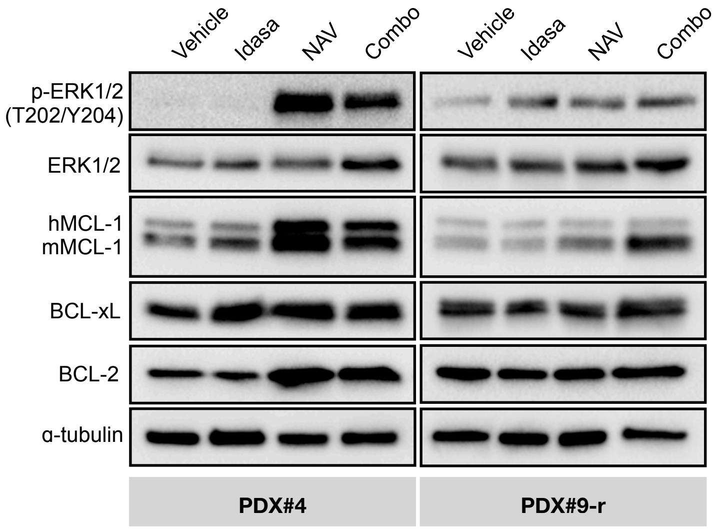

Supplementary Figure S13. ***In vivo*** **navitoclax exposure enhances RAS pathway activation and MCL-1 stabilization in PDX#4, but not PDX#9-r.** Steady-state pharmacodynamic analyses of splenocytes derived from PDX#4 and PDX#9-r treated for three consecutive days with vehicle, idasanutlin (30 mg/kg), navitoclax (50 mg/kg), or their combination by oral gavage (*n*=3 per treatment group). Immunoblots show: anti-apoptotic BCL-2 family proteins MCL-1, BCL-2, and BCL-xL; and phospho-ERK1/2 (T202/Y204) as an indicator of RAS pathway activation. m, mouse; h, human.

3. SUPPLEMENTARY TABLES AND LEGENDS

Supplementary Table S1. Primary (ALL) and patient-derived xenograft (PDX) samples

See Excel spreadsheet.

Supplementary Table S2. Antibodies used for flow cytometry and immunoblotting.

Antibody	Clone	Catalog no.	Manufacturer
**Antibodies for flow cytometry**			
anti-mCD45 PE-Cy7	30-F11	552848	BD Biosciences
anti-hCD45 APC-H7	2D1	560178	BD Biosciences
anti-hCD10 PE	HI10a	555375	BD Biosciences
anti-hCD19 APC	SJ25C1	561742	BD Biosciences
anti-hCD34 PerCP	8G12	340430	BD Biosciences
anti-CD7 APC	CD7-6B7	561604	BD Biosciences
anti-CD5 PerCP Cy5.5	L17F12	341109	BD Biosciences
**Antibodies for immunoblotting**			
α-tubulin	B-5-1-2	T6074	Sigma
BAK	D4E4	12105	CST
BAX	D2E11	5023	CST
BCL-2	D55G8	4223	CST
BCL-xL	54H6	2764	CST
BID	-	2002	CST
BIM	C34C5	2933	CST
cleaved PARP(Asp214)	D64E10	5625	CST
MCL-1	D2W9E	94296	CST
MDM2	IF2	MABE340	Merck Millipore
NOXA	D8L7U	14766	CST
p21	SX118	556430	BD Pharmingen
p53	DO-1	sc-126	Santa Cruz
p44/42 MAPK (Erk1/2)	137F5	4695	CST
Phospho-p44/42 MAPK (Erk1/2) (Thr202/Tyr204)	D12.14.4E	4370	CST
PUMA	D30C10	12450	CST

[a] m, mouse; h, human

[b] CST, Cell Signaling Technology

Supplementary Table S3. *TP53* mutations identified by targeted sequencing

Sample ID	Mutation
ALL#5-relapse	TP53:NM_000546.6:exon7:c.T721C:p.S241P
PDX#11-relapse	TP53:NM_000546.6:exon6:c.A659G:p.Y220C

Supplementary Table S4. Primary FDA-approved and clinically advanced drug idasanutlin combination screening in the relapsed NALM6 cell line

See Excel spreadsheet.

Supplementary Table S5. Combinatory primary FDA-approved library screening hit rates and quality control

Cell line	Experimental Condition	Mean Z' score	Mean robust Z' score	Hit rate (%)
NALM6	Single library drug	0.855	0.885	6.1
	+ Idasanutlin (IC_{25} [30 nM])	0.805	0.855	6.3

Raw screening data were analyzed with a script developed in the python programming language to determine quality control metrics and plate visualizations. Available at: https://github.com/hayden-bell/HTS-QC

Supplementary Table S6. Drug sensitivity of primary hits in the FDA-approved drug library idasanutlin combination screen

See Excel spreadsheet.

Supplementary Table S7. Clinically reported FDA-approved and clinically advanced drug parameters

See Excel spreadsheet.

Supplementary Table S8. Steady state plasma concentrations for oral administration of idasanutlin (30 mg/kg), navitoclax (50 mg/kg), and their combination in NSG mice. *N*=3 for each treatment group. Concentrations indicate mean ± s.d.

**Drug**	**Schedule**	**Route**	**Dose (mg/kg)**	**Conc. (µg/mL)**	**Conc. (µM)**
Idasanutlin	Monotherapy	p.o.	30	1.73 (±0.86)	2.81 (±1.39)
	Combination	p.o.	30	1.23 (±0.25)	1.99 (±0.40)
Navitoclax	Monotherapy	p.o.	50	0.88 (±0.64)	0.91 (±0.66)
	Combination	p.o.	50	1.58 (+0.17)	1.63 (±0.18)

Supplementary Table S9. Primer sequences and PCR reaction conditions for Sanger sequencing of *TP53* exons 4-8.

Target Region	Primer Sequences[a]	PCR reaction conditions[b]
TP53 exon 4	F 5'-CCTGGTCCTCTGACTGCTCT-3'<br>R 5'-GCCAGGCATTGAAGTCTCAT-3'	14 touchdown cycles + 20 standard cycles:<br>D 94°C 20s<br>A 57°C 60s<br>E 72°C 60s
TP53 exon 5	F 5'-GGATCCATCTGTTCACTTGTGCCCTG-3'<br>R 5'-GAATTCAACCAGCCCTGTCGTCTCTC-3'	14 touchdown cycles + 20 standard cycles:<br>D 94°C 20s<br>A 55°C 60s<br>E 72°C 60s
TP53 exon 6	F 5'-GCCTCTGATTCCTCACTGAT-3'<br>R 5'-GGAGGGCCACTGACAACCA-3'	14 touchdown cycles + 20 standard cycles:<br>D 94°C 20s<br>A 55°C 60s<br>E 72°C 60s
TP53 exon 7	F 5'-GGATCCAGGCGCACTGGCCTCATCTT-3'<br>R 5'-GAATTCAGGGGTCAGAGGCAAGCAGA-3'	14 touchdown cycles + 20 standard cycles:<br>D 94°C 20s<br>A 60°C 60s<br>E 72°C 60s
TP53 exon 8	F 5'-GAGCCTGGTTTTTTAAATGG-3'<br>R 5'-TTTGGCTGGGGAGAGGAGCT-3'	14 touchdown cycles + 20 standard cycles:<br>D 94°C 20s<br>A 60°C 60s<br>E 72°C 60s

[a] F, forward; R, reverse.

[b] initial denaturation was performed at 95°C for 10 minutes and the last cycle was followed by a final extension step at 72°C for 5 minutes in all cases. Touchdown cycles started at 7°C above annealing temperature and decreased by 0.5°C per cycle. D, denaturation; A, annealing; E, extension.

Supplementary Table S10. Target sequences of ON-TARGETplus Human siRNA, SMARTpool

Gene	Target Sequences (5’-3')
Non-targeting control (NTC)	1) UGGUUUACAUGUCGACUAA<br>2) UGGUUUACAUGUUGUGUGA<br>3) UGGUUUACAUGUUUUCUGA<br>4) UGGUUUACAUGUUUUCCUA
PMAIP1 (*NOXA)*	1) AAACUGAACUUCCGGCAGA<br>2) GAACCUGACUGCAUCAAAA<br>3) AAUCUGAUAUCCAAACUCU<br>4) GCAAGAACGCUCAACCGAG

4. SUPPLEMENTARY REFERENCES

1. Irving J, Matheson E, Minto L, Blair H, Case M, Halsey C, et al. Ras pathway mutations are prevalent in relapsed childhood acute lymphoblastic leukemia and confer sensitivity to MEK inhibition. Blood. 2014;124(23):3420-30.

2. Corsello S, Bittker J, Liu Z, Gould J, McCarren P, Hirschman J, et al. The Drug Repurposing Hub: a next-generation drug library and information resource. Nat Med. 2017;23:405-8.

3. Siramshetty V, Grishagin I, Nguyễn D, Peryea T, Skovpen Y, Stroganov O, et al. NCATS Inxight Drugs: a comprehensive and curated portal for translational research. Nuclei Acids Research. 2022;50(D1):D1307-D16.

4. Masic D, Fee K, Bell H, Case M, Witherington G, Lansbury S, et al. Hyperactive CREB subpopulations increase during therapy in pediatric B-lineage acute lymphoblastic leukemia. Haematologica. 2023;108(4):981-92.

5. Charan J, Kantharia N. How to calculate sample size in animal studies? J Pharmacol Pharmacother. 2013;4(4):303-6.

6. Nair A, Jacob S. A simple practice guide for dose conversion between animals and humans. J Basic Clin Pharm. 2016;7(2):27-31.

7. Bomken S, Buechler L, Rehe K, Ponthan F, Elder A, Blair H, et al. Lentiviral marking of patient-derived acute lymphoblastic leukaemic cells allows in vivo tracking of disease progression. Leukemia. 2013;27(3):718-21.

8. Kim S, Chen J, Cheng T, Gindulyte A, He J, He S, et al. PubChem 2023 update. Nuclei Acids Research. 2023;51(D1):D1373-D80.

9. Cao X, Deng X, May W. Cleavage of Bax to p18 Bax accelerates stress-induced apoptosis, and a cathepsin-like protease may rapidly degrade p18 Bax. Blood. 2003;102(7):2605-14.

10. Cartron P, Oliver L, Juin P, Meflah K, Vallette F. The p18 Truncated Form of Bax Behaves Like a Bcl-2 Homology Domain 3-only Protein. J Biol Chem. 2004;279(12):11504-12.
